# Supplementary material for: Continuous co-prescription of rebamipide prevents upper gastrointestinal bleeding in NSAID use for orthopaedic conditions: A nested case-control study using the LIFE Study database
Source: PLoS One. 2024 Jun 11;19(6):e0305320. doi: 10.1371/journal.pone.0305320 (PMC11166339; doi:10.1371/journal.pone.0305320)
Supplement: S1 Table — (DOCX) [file pone.0305320.s002.docx]

**S1 Table** ATC code list

| **Receipt code**  **Japan** | **ATC code**  **WHO** | **ATC 5th level name**  **WHO** | **Drug Classification Present study** | **Dose (mg) of NSAIDs**  **Japanese product** |
| --- | --- | --- | --- | --- |
| 620433401 | A02AB | Aluminium compounds | Other stomach medicine | _ |
| 620433501 | A02AB | Aluminium compounds | Other stomach medicine | _ |
| 620433601 | A02AB | Aluminium compounds | Other stomach medicine | _ |
| 620433701 | A02AB | Aluminium compounds | Other stomach medicine | _ |
| 620433913 | A02AB | Aluminium compounds | Other stomach medicine | _ |
| 620434101 | A02AB | Aluminium compounds | Other stomach medicine | _ |
| 620434501 | A02AB | Aluminium compounds | Other stomach medicine | _ |
| 620434601 | A02AB | Aluminium compounds | Other stomach medicine | _ |
| 620434801 | A02AB | Aluminium compounds | Other stomach medicine | _ |
| 621272501 | A02AB | Aluminium compounds | Other stomach medicine | _ |
| 620455501 | A02AD01 | Ordinary salt combinations | Other stomach medicine | _ |
| 620459901 | A02AD01 | Ordinary salt combinations | Other stomach medicine | _ |
| 621276601 | A02AD01 | Ordinary salt combinations | Other stomach medicine | _ |
| 612320182 | A02BA01 | Cimetidine | H2 blockers | _ |
| 612320183 | A02BA01 | Cimetidine | H2 blockers | _ |
| 612320399 | A02BA01 | Cimetidine | H2 blockers | _ |
| 620003446 | A02BA01 | Cimetidine | H2 blockers | _ |
| 620003527 | A02BA01 | Cimetidine | H2 blockers | _ |
| 620005980 | A02BA01 | Cimetidine | H2 blockers | _ |
| 620428807 | A02BA01 | Cimetidine | H2 blockers | _ |
| 620429707 | A02BA01 | Cimetidine | H2 blockers | _ |
| 620429720 | A02BA01 | Cimetidine | H2 blockers | _ |
| 620429734 | A02BA01 | Cimetidine | H2 blockers | _ |
| 620429739 | A02BA01 | Cimetidine | H2 blockers | _ |
| 620429744 | A02BA01 | Cimetidine | H2 blockers | _ |
| 620430303 | A02BA01 | Cimetidine | H2 blockers | _ |
| 621721101 | A02BA01 | Cimetidine | H2 blockers | _ |
| 621904001 | A02BA01 | Cimetidine | H2 blockers | _ |
| 621931501 | A02BA01 | Cimetidine | H2 blockers | _ |
| 621938801 | A02BA01 | Cimetidine | H2 blockers | _ |
| 621938901 | A02BA01 | Cimetidine | H2 blockers | _ |
| 621957702 | A02BA01 | Cimetidine | H2 blockers | _ |
| 621969602 | A02BA01 | Cimetidine | H2 blockers | _ |
| 622017601 | A02BA01 | Cimetidine | H2 blockers | _ |
| 622017701 | A02BA01 | Cimetidine | H2 blockers | _ |
| 622314600 | A02BA01 | Cimetidine | H2 blockers | _ |
| 622732400 | A02BA01 | Cimetidine | H2 blockers | _ |
| 610453144 | A02BA02 | Ranitidine | H2 blockers | _ |
| 612320531 | A02BA02 | Ranitidine | H2 blockers | _ |
| 620000042 | A02BA02 | Ranitidine | H2 blockers | _ |
| 620009448 | A02BA02 | Ranitidine | H2 blockers | _ |
| 620009449 | A02BA02 | Ranitidine | H2 blockers | _ |
| 621408401 | A02BA02 | Ranitidine | H2 blockers | _ |
| 621408501 | A02BA02 | Ranitidine | H2 blockers | _ |
| 621408702 | A02BA02 | Ranitidine | H2 blockers | _ |
| 621408801 | A02BA02 | Ranitidine | H2 blockers | _ |
| 621408901 | A02BA02 | Ranitidine | H2 blockers | _ |
| 621409102 | A02BA02 | Ranitidine | H2 blockers | _ |
| 621409301 | A02BA02 | Ranitidine | H2 blockers | _ |
| 621409401 | A02BA02 | Ranitidine | H2 blockers | _ |
| 621409601 | A02BA02 | Ranitidine | H2 blockers | _ |
| 621409701 | A02BA02 | Ranitidine | H2 blockers | _ |
| 621473401 | A02BA02 | Ranitidine | H2 blockers | _ |
| 621688001 | A02BA02 | Ranitidine | H2 blockers | _ |
| 621970001 | A02BA02 | Ranitidine | H2 blockers | _ |
| 622732500 | A02BA02 | Ranitidine | H2 blockers | _ |
| 622732600 | A02BA02 | Ranitidine | H2 blockers | _ |
| 610406079 | A02BA03 | Famotidine | H2 blockers | _ |
| 610444041 | A02BA03 | Famotidine | H2 blockers | _ |
| 610444042 | A02BA03 | Famotidine | H2 blockers | _ |
| 610463106 | A02BA03 | Famotidine | H2 blockers | _ |
| 610463107 | A02BA03 | Famotidine | H2 blockers | _ |
| 610463165 | A02BA03 | Famotidine | H2 blockers | _ |
| 610463166 | A02BA03 | Famotidine | H2 blockers | _ |
| 610463167 | A02BA03 | Famotidine | H2 blockers | _ |
| 612320349 | A02BA03 | Famotidine | H2 blockers | _ |
| 612320350 | A02BA03 | Famotidine | H2 blockers | _ |
| 612320417 | A02BA03 | Famotidine | H2 blockers | _ |
| 620000128 | A02BA03 | Famotidine | H2 blockers | _ |
| 620000129 | A02BA03 | Famotidine | H2 blockers | _ |
| 620002098 | A02BA03 | Famotidine | H2 blockers | _ |
| 620003601 | A02BA03 | Famotidine | H2 blockers | _ |
| 620004032 | A02BA03 | Famotidine | H2 blockers | _ |
| 620004035 | A02BA03 | Famotidine | H2 blockers | _ |
| 620004036 | A02BA03 | Famotidine | H2 blockers | _ |
| 620005527 | A02BA03 | Famotidine | H2 blockers | _ |
| 620005528 | A02BA03 | Famotidine | H2 blockers | _ |
| 620006751 | A02BA03 | Famotidine | H2 blockers | _ |
| 620008501 | A02BA03 | Famotidine | H2 blockers | _ |
| 620008502 | A02BA03 | Famotidine | H2 blockers | _ |
| 621473502 | A02BA03 | Famotidine | H2 blockers | _ |
| 621473603 | A02BA03 | Famotidine | H2 blockers | _ |
| 621473802 | A02BA03 | Famotidine | H2 blockers | _ |
| 621473901 | A02BA03 | Famotidine | H2 blockers | _ |
| 621474102 | A02BA03 | Famotidine | H2 blockers | _ |
| 621474302 | A02BA03 | Famotidine | H2 blockers | _ |
| 621474401 | A02BA03 | Famotidine | H2 blockers | _ |
| 621474502 | A02BA03 | Famotidine | H2 blockers | _ |
| 621474601 | A02BA03 | Famotidine | H2 blockers | _ |
| 621474702 | A02BA03 | Famotidine | H2 blockers | _ |
| 621474801 | A02BA03 | Famotidine | H2 blockers | _ |
| 621474901 | A02BA03 | Famotidine | H2 blockers | _ |
| 621475002 | A02BA03 | Famotidine | H2 blockers | _ |
| 621475102 | A02BA03 | Famotidine | H2 blockers | _ |
| 621475302 | A02BA03 | Famotidine | H2 blockers | _ |
| 621475401 | A02BA03 | Famotidine | H2 blockers | _ |
| 621475502 | A02BA03 | Famotidine | H2 blockers | _ |
| 621475601 | A02BA03 | Famotidine | H2 blockers | _ |
| 621475702 | A02BA03 | Famotidine | H2 blockers | _ |
| 621475801 | A02BA03 | Famotidine | H2 blockers | _ |
| 621475901 | A02BA03 | Famotidine | H2 blockers | _ |
| 621476002 | A02BA03 | Famotidine | H2 blockers | _ |
| 621476101 | A02BA03 | Famotidine | H2 blockers | _ |
| 621523003 | A02BA03 | Famotidine | H2 blockers | _ |
| 621558203 | A02BA03 | Famotidine | H2 blockers | _ |
| 621558303 | A02BA03 | Famotidine | H2 blockers | _ |
| 621559101 | A02BA03 | Famotidine | H2 blockers | _ |
| 621559201 | A02BA03 | Famotidine | H2 blockers | _ |
| 621622001 | A02BA03 | Famotidine | H2 blockers | _ |
| 621622101 | A02BA03 | Famotidine | H2 blockers | _ |
| 621631201 | A02BA03 | Famotidine | H2 blockers | _ |
| 621631301 | A02BA03 | Famotidine | H2 blockers | _ |
| 621631402 | A02BA03 | Famotidine | H2 blockers | _ |
| 621631701 | A02BA03 | Famotidine | H2 blockers | _ |
| 621638202 | A02BA03 | Famotidine | H2 blockers | _ |
| 621638401 | A02BA03 | Famotidine | H2 blockers | _ |
| 621638501 | A02BA03 | Famotidine | H2 blockers | _ |
| 621638602 | A02BA03 | Famotidine | H2 blockers | _ |
| 621639601 | A02BA03 | Famotidine | H2 blockers | _ |
| 621642601 | A02BA03 | Famotidine | H2 blockers | _ |
| 621642701 | A02BA03 | Famotidine | H2 blockers | _ |
| 621681501 | A02BA03 | Famotidine | H2 blockers | _ |
| 621681601 | A02BA03 | Famotidine | H2 blockers | _ |
| 621687102 | A02BA03 | Famotidine | H2 blockers | _ |
| 621687202 | A02BA03 | Famotidine | H2 blockers | _ |
| 621687301 | A02BA03 | Famotidine | H2 blockers | _ |
| 621687401 | A02BA03 | Famotidine | H2 blockers | _ |
| 621743003 | A02BA03 | Famotidine | H2 blockers | _ |
| 621748803 | A02BA03 | Famotidine | H2 blockers | _ |
| 621863401 | A02BA03 | Famotidine | H2 blockers | _ |
| 621863501 | A02BA03 | Famotidine | H2 blockers | _ |
| 621941101 | A02BA03 | Famotidine | H2 blockers | _ |
| 622013102 | A02BA03 | Famotidine | H2 blockers | _ |
| 622037501 | A02BA03 | Famotidine | H2 blockers | _ |
| 622263801 | A02BA03 | Famotidine | H2 blockers | _ |
| 622264801 | A02BA03 | Famotidine | H2 blockers | _ |
| 622321500 | A02BA03 | Famotidine | H2 blockers | _ |
| 622604501 | A02BA03 | Famotidine | H2 blockers | _ |
| 622606801 | A02BA03 | Famotidine | H2 blockers | _ |
| 622732700 | A02BA03 | Famotidine | H2 blockers | _ |
| 622732800 | A02BA03 | Famotidine | H2 blockers | _ |
| 622732900 | A02BA03 | Famotidine | H2 blockers | _ |
| 622733000 | A02BA03 | Famotidine | H2 blockers | _ |
| 620005869 | A02BA04 | Nizatidine | H2 blockers | _ |
| 620005870 | A02BA04 | Nizatidine | H2 blockers | _ |
| 620008032 | A02BA04 | Nizatidine | H2 blockers | _ |
| 620008033 | A02BA04 | Nizatidine | H2 blockers | _ |
| 621476202 | A02BA04 | Nizatidine | H2 blockers | _ |
| 621476301 | A02BA04 | Nizatidine | H2 blockers | _ |
| 621476901 | A02BA04 | Nizatidine | H2 blockers | _ |
| 621477102 | A02BA04 | Nizatidine | H2 blockers | _ |
| 621477201 | A02BA04 | Nizatidine | H2 blockers | _ |
| 621477301 | A02BA04 | Nizatidine | H2 blockers | _ |
| 621526902 | A02BA04 | Nizatidine | H2 blockers | _ |
| 621527102 | A02BA04 | Nizatidine | H2 blockers | _ |
| 622733300 | A02BA04 | Nizatidine | H2 blockers | _ |
| 622733400 | A02BA04 | Nizatidine | H2 blockers | _ |
| 622733500 | A02BA04 | Nizatidine | H2 blockers | _ |
| 620431502 | A02BA06 | Roxatidine | H2 blockers | _ |
| 620431602 | A02BA06 | Roxatidine | H2 blockers | _ |
| 621694301 | A02BA06 | Roxatidine | H2 blockers | _ |
| 621694401 | A02BA06 | Roxatidine | H2 blockers | _ |
| 621694801 | A02BA06 | Roxatidine | H2 blockers | _ |
| 621891201 | A02BA06 | Roxatidine | H2 blockers | _ |
| 622136601 | A02BA06 | Roxatidine | H2 blockers | _ |
| 622733100 | A02BA06 | Roxatidine | H2 blockers | _ |
| 622733200 | A02BA06 | Roxatidine | H2 blockers | _ |
| 610443006 | A02BA08 | Lafutidine | H2 blockers | _ |
| 610443007 | A02BA08 | Lafutidine | H2 blockers | _ |
| 622135001 | A02BA08 | Lafutidine | H2 blockers | _ |
| 622135101 | A02BA08 | Lafutidine | H2 blockers | _ |
| 622185601 | A02BA08 | Lafutidine | H2 blockers | _ |
| 622185701 | A02BA08 | Lafutidine | H2 blockers | _ |
| 622188601 | A02BA08 | Lafutidine | H2 blockers | _ |
| 622188701 | A02BA08 | Lafutidine | H2 blockers | _ |
| 622194501 | A02BA08 | Lafutidine | H2 blockers | _ |
| 622194601 | A02BA08 | Lafutidine | H2 blockers | _ |
| 622197101 | A02BA08 | Lafutidine | H2 blockers | _ |
| 622197201 | A02BA08 | Lafutidine | H2 blockers | _ |
| 622204601 | A02BA08 | Lafutidine | H2 blockers | _ |
| 622204701 | A02BA08 | Lafutidine | H2 blockers | _ |
| 622205901 | A02BA08 | Lafutidine | H2 blockers | _ |
| 622206001 | A02BA08 | Lafutidine | H2 blockers | _ |
| 622208501 | A02BA08 | Lafutidine | H2 blockers | _ |
| 622208601 | A02BA08 | Lafutidine | H2 blockers | _ |
| 622219301 | A02BA08 | Lafutidine | H2 blockers | _ |
| 622219401 | A02BA08 | Lafutidine | H2 blockers | _ |
| 622221601 | A02BA08 | Lafutidine | H2 blockers | _ |
| 622221701 | A02BA08 | Lafutidine | H2 blockers | _ |
| 610409353 | A02BB01 | Misoprostol | Misoprostol | _ |
| 612320553 | A02BB01 | Misoprostol | Misoprostol | _ |
| 622404401 | A02BC | Proton pump inhibitors | Proton pump inhibitor | _ |
| 622404501 | A02BC | Proton pump inhibitors | Proton pump inhibitor | _ |
| 610443068 | A02BC01 | Omeprazole | Proton pump inhibitor | _ |
| 610443069 | A02BC01 | Omeprazole | Proton pump inhibitor | _ |
| 610443070 | A02BC01 | Omeprazole | Proton pump inhibitor | _ |
| 610443071 | A02BC01 | Omeprazole | Proton pump inhibitor | _ |
| 620001983 | A02BC01 | Omeprazole | Proton pump inhibitor | _ |
| 620001984 | A02BC01 | Omeprazole | Proton pump inhibitor | _ |
| 620001985 | A02BC01 | Omeprazole | Proton pump inhibitor | _ |
| 620001986 | A02BC01 | Omeprazole | Proton pump inhibitor | _ |
| 620002694 | A02BC01 | Omeprazole | Proton pump inhibitor | _ |
| 620002695 | A02BC01 | Omeprazole | Proton pump inhibitor | _ |
| 620003914 | A02BC01 | Omeprazole | Proton pump inhibitor | _ |
| 621622303 | A02BC01 | Omeprazole | Proton pump inhibitor | _ |
| 621622403 | A02BC01 | Omeprazole | Proton pump inhibitor | _ |
| 621630201 | A02BC01 | Omeprazole | Proton pump inhibitor | _ |
| 621630301 | A02BC01 | Omeprazole | Proton pump inhibitor | _ |
| 621630502 | A02BC01 | Omeprazole | Proton pump inhibitor | _ |
| 621630601 | A02BC01 | Omeprazole | Proton pump inhibitor | _ |
| 621630701 | A02BC01 | Omeprazole | Proton pump inhibitor | _ |
| 621680901 | A02BC01 | Omeprazole | Proton pump inhibitor | _ |
| 621681001 | A02BC01 | Omeprazole | Proton pump inhibitor | _ |
| 621681401 | A02BC01 | Omeprazole | Proton pump inhibitor | _ |
| 621794301 | A02BC01 | Omeprazole | Proton pump inhibitor | _ |
| 621977902 | A02BC01 | Omeprazole | Proton pump inhibitor | _ |
| 622077801 | A02BC01 | Omeprazole | Proton pump inhibitor | _ |
| 622735200 | A02BC01 | Omeprazole | Proton pump inhibitor | _ |
| 622735300 | A02BC01 | Omeprazole | Proton pump inhibitor | _ |
| 610462010 | A02BC03 | Lansoprazole | Proton pump inhibitor | _ |
| 610462011 | A02BC03 | Lansoprazole | Proton pump inhibitor | _ |
| 612320549 | A02BC03 | Lansoprazole | Proton pump inhibitor | _ |
| 612320550 | A02BC03 | Lansoprazole | Proton pump inhibitor | _ |
| 620002749 | A02BC03 | Lansoprazole | Proton pump inhibitor | _ |
| 620002750 | A02BC03 | Lansoprazole | Proton pump inhibitor | _ |
| 620002871 | A02BC03 | Lansoprazole | Proton pump inhibitor | _ |
| 620002872 | A02BC03 | Lansoprazole | Proton pump inhibitor | _ |
| 620005581 | A02BC03 | Lansoprazole | Proton pump inhibitor | _ |
| 620005583 | A02BC03 | Lansoprazole | Proton pump inhibitor | _ |
| 620005584 | A02BC03 | Lansoprazole | Proton pump inhibitor | _ |
| 620005585 | A02BC03 | Lansoprazole | Proton pump inhibitor | _ |
| 620007127 | A02BC03 | Lansoprazole | Proton pump inhibitor | _ |
| 620007128 | A02BC03 | Lansoprazole | Proton pump inhibitor | _ |
| 620009451 | A02BC03 | Lansoprazole | Proton pump inhibitor | _ |
| 620009452 | A02BC03 | Lansoprazole | Proton pump inhibitor | _ |
| 621666205 | A02BC03 | Lansoprazole | Proton pump inhibitor | _ |
| 621666305 | A02BC03 | Lansoprazole | Proton pump inhibitor | _ |
| 621673701 | A02BC03 | Lansoprazole | Proton pump inhibitor | _ |
| 621673801 | A02BC03 | Lansoprazole | Proton pump inhibitor | _ |
| 621693101 | A02BC03 | Lansoprazole | Proton pump inhibitor | _ |
| 621693201 | A02BC03 | Lansoprazole | Proton pump inhibitor | _ |
| 621743701 | A02BC03 | Lansoprazole | Proton pump inhibitor | _ |
| 621743801 | A02BC03 | Lansoprazole | Proton pump inhibitor | _ |
| 621780303 | A02BC03 | Lansoprazole | Proton pump inhibitor | _ |
| 621780403 | A02BC03 | Lansoprazole | Proton pump inhibitor | _ |
| 621796301 | A02BC03 | Lansoprazole | Proton pump inhibitor | _ |
| 621796401 | A02BC03 | Lansoprazole | Proton pump inhibitor | _ |
| 621919001 | A02BC03 | Lansoprazole | Proton pump inhibitor | _ |
| 621919101 | A02BC03 | Lansoprazole | Proton pump inhibitor | _ |
| 622015801 | A02BC03 | Lansoprazole | Proton pump inhibitor | _ |
| 622015901 | A02BC03 | Lansoprazole | Proton pump inhibitor | _ |
| 622023101 | A02BC03 | Lansoprazole | Proton pump inhibitor | _ |
| 622023201 | A02BC03 | Lansoprazole | Proton pump inhibitor | _ |
| 622034601 | A02BC03 | Lansoprazole | Proton pump inhibitor | _ |
| 622034701 | A02BC03 | Lansoprazole | Proton pump inhibitor | _ |
| 622469801 | A02BC03 | Lansoprazole | Proton pump inhibitor | _ |
| 622469901 | A02BC03 | Lansoprazole | Proton pump inhibitor | _ |
| 622617200 | A02BC03 | Lansoprazole | Proton pump inhibitor | _ |
| 622735400 | A02BC03 | Lansoprazole | Proton pump inhibitor | _ |
| 622735500 | A02BC03 | Lansoprazole | Proton pump inhibitor | _ |
| 622846800 | A02BC03 | Lansoprazole | Proton pump inhibitor | _ |
| 610412202 | A02BC04 | Rabeprazole | Proton pump inhibitor | _ |
| 610412203 | A02BC04 | Rabeprazole | Proton pump inhibitor | _ |
| 621997301 | A02BC04 | Rabeprazole | Proton pump inhibitor | _ |
| 621999501 | A02BC04 | Rabeprazole | Proton pump inhibitor | _ |
| 621999601 | A02BC04 | Rabeprazole | Proton pump inhibitor | _ |
| 621999901 | A02BC04 | Rabeprazole | Proton pump inhibitor | _ |
| 622000001 | A02BC04 | Rabeprazole | Proton pump inhibitor | _ |
| 622002201 | A02BC04 | Rabeprazole | Proton pump inhibitor | _ |
| 622002301 | A02BC04 | Rabeprazole | Proton pump inhibitor | _ |
| 622005301 | A02BC04 | Rabeprazole | Proton pump inhibitor | _ |
| 622005401 | A02BC04 | Rabeprazole | Proton pump inhibitor | _ |
| 622007301 | A02BC04 | Rabeprazole | Proton pump inhibitor | _ |
| 622007401 | A02BC04 | Rabeprazole | Proton pump inhibitor | _ |
| 622011201 | A02BC04 | Rabeprazole | Proton pump inhibitor | _ |
| 622011301 | A02BC04 | Rabeprazole | Proton pump inhibitor | _ |
| 622012901 | A02BC04 | Rabeprazole | Proton pump inhibitor | _ |
| 622013001 | A02BC04 | Rabeprazole | Proton pump inhibitor | _ |
| 622016201 | A02BC04 | Rabeprazole | Proton pump inhibitor | _ |
| 622016301 | A02BC04 | Rabeprazole | Proton pump inhibitor | _ |
| 622020603 | A02BC04 | Rabeprazole | Proton pump inhibitor | _ |
| 622020703 | A02BC04 | Rabeprazole | Proton pump inhibitor | _ |
| 622023301 | A02BC04 | Rabeprazole | Proton pump inhibitor | _ |
| 622023401 | A02BC04 | Rabeprazole | Proton pump inhibitor | _ |
| 622025601 | A02BC04 | Rabeprazole | Proton pump inhibitor | _ |
| 622025701 | A02BC04 | Rabeprazole | Proton pump inhibitor | _ |
| 622026001 | A02BC04 | Rabeprazole | Proton pump inhibitor | _ |
| 622026101 | A02BC04 | Rabeprazole | Proton pump inhibitor | _ |
| 622031601 | A02BC04 | Rabeprazole | Proton pump inhibitor | _ |
| 622031701 | A02BC04 | Rabeprazole | Proton pump inhibitor | _ |
| 622032801 | A02BC04 | Rabeprazole | Proton pump inhibitor | _ |
| 622032901 | A02BC04 | Rabeprazole | Proton pump inhibitor | _ |
| 622035501 | A02BC04 | Rabeprazole | Proton pump inhibitor | _ |
| 622035601 | A02BC04 | Rabeprazole | Proton pump inhibitor | _ |
| 622040101 | A02BC04 | Rabeprazole | Proton pump inhibitor | _ |
| 622040201 | A02BC04 | Rabeprazole | Proton pump inhibitor | _ |
| 622060201 | A02BC04 | Rabeprazole | Proton pump inhibitor | _ |
| 622060301 | A02BC04 | Rabeprazole | Proton pump inhibitor | _ |
| 622089501 | A02BC04 | Rabeprazole | Proton pump inhibitor | _ |
| 622089601 | A02BC04 | Rabeprazole | Proton pump inhibitor | _ |
| 622118601 | A02BC04 | Rabeprazole | Proton pump inhibitor | _ |
| 622118701 | A02BC04 | Rabeprazole | Proton pump inhibitor | _ |
| 622402601 | A02BC04 | Rabeprazole | Proton pump inhibitor | _ |
| 622505501 | A02BC04 | Rabeprazole | Proton pump inhibitor | _ |
| 622505601 | A02BC04 | Rabeprazole | Proton pump inhibitor | _ |
| 622617000 | A02BC04 | Rabeprazole | Proton pump inhibitor | _ |
| 622617100 | A02BC04 | Rabeprazole | Proton pump inhibitor | _ |
| 622702001 | A02BC04 | Rabeprazole | Proton pump inhibitor | _ |
| 622702101 | A02BC04 | Rabeprazole | Proton pump inhibitor | _ |
| 622784501 | A02BC04 | Rabeprazole | Proton pump inhibitor | _ |
| 622785701 | A02BC04 | Rabeprazole | Proton pump inhibitor | _ |
| 622787001 | A02BC04 | Rabeprazole | Proton pump inhibitor | _ |
| 622789801 | A02BC04 | Rabeprazole | Proton pump inhibitor | _ |
| 622791001 | A02BC04 | Rabeprazole | Proton pump inhibitor | _ |
| 622793401 | A02BC04 | Rabeprazole | Proton pump inhibitor | _ |
| 622794001 | A02BC04 | Rabeprazole | Proton pump inhibitor | _ |
| 622794501 | A02BC04 | Rabeprazole | Proton pump inhibitor | _ |
| 622795701 | A02BC04 | Rabeprazole | Proton pump inhibitor | _ |
| 622798401 | A02BC04 | Rabeprazole | Proton pump inhibitor | _ |
| 622800501 | A02BC04 | Rabeprazole | Proton pump inhibitor | _ |
| 622801201 | A02BC04 | Rabeprazole | Proton pump inhibitor | _ |
| 622805901 | A02BC04 | Rabeprazole | Proton pump inhibitor | _ |
| 622813901 | A02BC04 | Rabeprazole | Proton pump inhibitor | _ |
| 622815801 | A02BC04 | Rabeprazole | Proton pump inhibitor | _ |
| 622816501 | A02BC04 | Rabeprazole | Proton pump inhibitor | _ |
| 622816801 | A02BC04 | Rabeprazole | Proton pump inhibitor | _ |
| 622829201 | A02BC04 | Rabeprazole | Proton pump inhibitor | _ |
| 622829301 | A02BC04 | Rabeprazole | Proton pump inhibitor | _ |
| 622846900 | A02BC04 | Rabeprazole | Proton pump inhibitor | _ |
| 622080701 | A02BC05 | Esomeprazole | Proton pump inhibitor | _ |
| 622080801 | A02BC05 | Esomeprazole | Proton pump inhibitor | _ |
| 622624801 | A02BC05 | Esomeprazole | Proton pump inhibitor | _ |
| 622624901 | A02BC05 | Esomeprazole | Proton pump inhibitor | _ |
| 620000147 | A02BX | Other drugs for peptic ulcer and gastro-oesophageal reflux disease (GORD) | Rebamipide | _ |
| 620009460 | A02BX | Other drugs for peptic ulcer and gastro-oesophageal reflux disease (GORD) | Rebamipide | _ |
| 620009461 | A02BX | Other drugs for peptic ulcer and gastro-oesophageal reflux disease (GORD) | Rebamipide | _ |
| 620009462 | A02BX | Other drugs for peptic ulcer and gastro-oesophageal reflux disease (GORD) | Rebamipide | _ |
| 620009463 | A02BX | Other drugs for peptic ulcer and gastro-oesophageal reflux disease (GORD) | Rebamipide | _ |
| 620009465 | A02BX | Other drugs for peptic ulcer and gastro-oesophageal reflux disease (GORD) | Rebamipide | _ |
| 620009466 | A02BX | Other drugs for peptic ulcer and gastro-oesophageal reflux disease (GORD) | Rebamipide | _ |
| 620452801 | A02BX | Other drugs for peptic ulcer and gastro-oesophageal reflux disease (GORD) | Rebamipide | _ |
| 621902902 | A02BX | Other drugs for peptic ulcer and gastro-oesophageal reflux disease (GORD) | Rebamipide | _ |
| 621930401 | A02BX | Other drugs for peptic ulcer and gastro-oesophageal reflux disease (GORD) | Rebamipide | _ |
| 621937901 | A02BX | Other drugs for peptic ulcer and gastro-oesophageal reflux disease (GORD) | Rebamipide | _ |
| 621938001 | A02BX | Other drugs for peptic ulcer and gastro-oesophageal reflux disease (GORD) | Rebamipide | _ |
| 621941001 | A02BX | Other drugs for peptic ulcer and gastro-oesophageal reflux disease (GORD) | Rebamipide | _ |
| 621941801 | A02BX | Other drugs for peptic ulcer and gastro-oesophageal reflux disease (GORD) | Rebamipide | _ |
| 621944501 | A02BX | Other drugs for peptic ulcer and gastro-oesophageal reflux disease (GORD) | Rebamipide | _ |
| 621946001 | A02BX | Other drugs for peptic ulcer and gastro-oesophageal reflux disease (GORD) | Rebamipide | _ |
| 621949701 | A02BX | Other drugs for peptic ulcer and gastro-oesophageal reflux disease (GORD) | Rebamipide | _ |
| 621949801 | A02BX | Other drugs for peptic ulcer and gastro-oesophageal reflux disease (GORD) | Rebamipide | _ |
| 621950301 | A02BX | Other drugs for peptic ulcer and gastro-oesophageal reflux disease (GORD) | Rebamipide | _ |
| 621952801 | A02BX | Other drugs for peptic ulcer and gastro-oesophageal reflux disease (GORD) | Rebamipide | _ |
| 621952901 | A02BX | Other drugs for peptic ulcer and gastro-oesophageal reflux disease (GORD) | Rebamipide | _ |
| 621956501 | A02BX | Other drugs for peptic ulcer and gastro-oesophageal reflux disease (GORD) | Rebamipide | _ |
| 621965701 | A02BX | Other drugs for peptic ulcer and gastro-oesophageal reflux disease (GORD) | Rebamipide | _ |
| 621965801 | A02BX | Other drugs for peptic ulcer and gastro-oesophageal reflux disease (GORD) | Rebamipide | _ |
| 621973901 | A02BX | Other drugs for peptic ulcer and gastro-oesophageal reflux disease (GORD) | Rebamipide | _ |
| 621981601 | A02BX | Other drugs for peptic ulcer and gastro-oesophageal reflux disease (GORD) | Rebamipide | _ |
| 621986701 | A02BX | Other drugs for peptic ulcer and gastro-oesophageal reflux disease (GORD) | Rebamipide | _ |
| 621989101 | A02BX | Other drugs for peptic ulcer and gastro-oesophageal reflux disease (GORD) | Rebamipide | _ |
| 621993701 | A02BX | Other drugs for peptic ulcer and gastro-oesophageal reflux disease (GORD) | Rebamipide | _ |
| 622089701 | A02BX | Other drugs for peptic ulcer and gastro-oesophageal reflux disease (GORD) | Rebamipide | _ |
| 622400601 | A02BX | Other drugs for peptic ulcer and gastro-oesophageal reflux disease (GORD) | Rebamipide | _ |
| 622454101 | A02BX | Other drugs for peptic ulcer and gastro-oesophageal reflux disease (GORD) | Rebamipide | _ |
| 610406170 | A02BX | Other drugs for peptic ulcer and gastro-oesophageal reflux disease (GORD) | Other stomach medicine | _ |
| 610406389 | A02BX | Other drugs for peptic ulcer and gastro-oesophageal reflux disease (GORD) | Other stomach medicine | _ |
| 610412101 | A02BX | Other drugs for peptic ulcer and gastro-oesophageal reflux disease (GORD) | Other stomach medicine | _ |
| 610422181 | A02BX | Other drugs for peptic ulcer and gastro-oesophageal reflux disease (GORD) | Other stomach medicine | _ |
| 610444152 | A02BX | Other drugs for peptic ulcer and gastro-oesophageal reflux disease (GORD) | Other stomach medicine | _ |
| 610454047 | A02BX | Other drugs for peptic ulcer and gastro-oesophageal reflux disease (GORD) | Other stomach medicine | _ |
| 610463146 | A02BX | Other drugs for peptic ulcer and gastro-oesophageal reflux disease (GORD) | Other stomach medicine | _ |
| 612320346 | A02BX | Other drugs for peptic ulcer and gastro-oesophageal reflux disease (GORD) | Other stomach medicine | _ |
| 612320347 | A02BX | Other drugs for peptic ulcer and gastro-oesophageal reflux disease (GORD) | Other stomach medicine | _ |
| 612320436 | A02BX | Other drugs for peptic ulcer and gastro-oesophageal reflux disease (GORD) | Other stomach medicine | _ |
| 612320437 | A02BX | Other drugs for peptic ulcer and gastro-oesophageal reflux disease (GORD) | Other stomach medicine | _ |
| 612320438 | A02BX | Other drugs for peptic ulcer and gastro-oesophageal reflux disease (GORD) | Other stomach medicine | _ |
| 620002058 | A02BX | Other drugs for peptic ulcer and gastro-oesophageal reflux disease (GORD) | Other stomach medicine | _ |
| 620003533 | A02BX | Other drugs for peptic ulcer and gastro-oesophageal reflux disease (GORD) | Other stomach medicine | _ |
| 620004044 | A02BX | Other drugs for peptic ulcer and gastro-oesophageal reflux disease (GORD) | Other stomach medicine | _ |
| 620005368 | A02BX | Other drugs for peptic ulcer and gastro-oesophageal reflux disease (GORD) | Other stomach medicine | _ |
| 620006034 | A02BX | Other drugs for peptic ulcer and gastro-oesophageal reflux disease (GORD) | Other stomach medicine | _ |
| 620006035 | A02BX | Other drugs for peptic ulcer and gastro-oesophageal reflux disease (GORD) | Other stomach medicine | _ |
| 620006856 | A02BX | Other drugs for peptic ulcer and gastro-oesophageal reflux disease (GORD) | Other stomach medicine | _ |
| 620007935 | A02BX | Other drugs for peptic ulcer and gastro-oesophageal reflux disease (GORD) | Other stomach medicine | _ |
| 620007936 | A02BX | Other drugs for peptic ulcer and gastro-oesophageal reflux disease (GORD) | Other stomach medicine | _ |
| 620007942 | A02BX | Other drugs for peptic ulcer and gastro-oesophageal reflux disease (GORD) | Other stomach medicine | _ |
| 620008078 | A02BX | Other drugs for peptic ulcer and gastro-oesophageal reflux disease (GORD) | Other stomach medicine | _ |
| 620008079 | A02BX | Other drugs for peptic ulcer and gastro-oesophageal reflux disease (GORD) | Other stomach medicine | _ |
| 620008080 | A02BX | Other drugs for peptic ulcer and gastro-oesophageal reflux disease (GORD) | Other stomach medicine | _ |
| 620008082 | A02BX | Other drugs for peptic ulcer and gastro-oesophageal reflux disease (GORD) | Other stomach medicine | _ |
| 620008085 | A02BX | Other drugs for peptic ulcer and gastro-oesophageal reflux disease (GORD) | Other stomach medicine | _ |
| 620008672 | A02BX | Other drugs for peptic ulcer and gastro-oesophageal reflux disease (GORD) | Other stomach medicine | _ |
| 620008740 | A02BX | Other drugs for peptic ulcer and gastro-oesophageal reflux disease (GORD) | Other stomach medicine | _ |
| 620009347 | A02BX | Other drugs for peptic ulcer and gastro-oesophageal reflux disease (GORD) | Other stomach medicine | _ |
| 620009450 | A02BX | Other drugs for peptic ulcer and gastro-oesophageal reflux disease (GORD) | Other stomach medicine | _ |
| 620445701 | A02BX | Other drugs for peptic ulcer and gastro-oesophageal reflux disease (GORD) | Other stomach medicine | _ |
| 620445802 | A02BX | Other drugs for peptic ulcer and gastro-oesophageal reflux disease (GORD) | Other stomach medicine | _ |
| 620446001 | A02BX | Other drugs for peptic ulcer and gastro-oesophageal reflux disease (GORD) | Other stomach medicine | _ |
| 620446204 | A02BX | Other drugs for peptic ulcer and gastro-oesophageal reflux disease (GORD) | Other stomach medicine | _ |
| 620446205 | A02BX | Other drugs for peptic ulcer and gastro-oesophageal reflux disease (GORD) | Other stomach medicine | _ |
| 620447001 | A02BX | Other drugs for peptic ulcer and gastro-oesophageal reflux disease (GORD) | Other stomach medicine | _ |
| 620447402 | A02BX | Other drugs for peptic ulcer and gastro-oesophageal reflux disease (GORD) | Other stomach medicine | _ |
| 620447601 | A02BX | Other drugs for peptic ulcer and gastro-oesophageal reflux disease (GORD) | Other stomach medicine | _ |
| 620447701 | A02BX | Other drugs for peptic ulcer and gastro-oesophageal reflux disease (GORD) | Other stomach medicine | _ |
| 620447704 | A02BX | Other drugs for peptic ulcer and gastro-oesophageal reflux disease (GORD) | Other stomach medicine | _ |
| 620448401 | A02BX | Other drugs for peptic ulcer and gastro-oesophageal reflux disease (GORD) | Other stomach medicine | _ |
| 620452104 | A02BX | Other drugs for peptic ulcer and gastro-oesophageal reflux disease (GORD) | Other stomach medicine | _ |
| 620452603 | A02BX | Other drugs for peptic ulcer and gastro-oesophageal reflux disease (GORD) | Other stomach medicine | _ |
| 620457501 | A02BX | Other drugs for peptic ulcer and gastro-oesophageal reflux disease (GORD) | Other stomach medicine | _ |
| 620459001 | A02BX | Other drugs for peptic ulcer and gastro-oesophageal reflux disease (GORD) | Other stomach medicine | _ |
| 620459201 | A02BX | Other drugs for peptic ulcer and gastro-oesophageal reflux disease (GORD) | Other stomach medicine | _ |
| 621274801 | A02BX | Other drugs for peptic ulcer and gastro-oesophageal reflux disease (GORD) | Other stomach medicine | _ |
| 621275001 | A02BX | Other drugs for peptic ulcer and gastro-oesophageal reflux disease (GORD) | Other stomach medicine | _ |
| 621276001 | A02BX | Other drugs for peptic ulcer and gastro-oesophageal reflux disease (GORD) | Other stomach medicine | _ |
| 621276201 | A02BX | Other drugs for peptic ulcer and gastro-oesophageal reflux disease (GORD) | Other stomach medicine | _ |
| 621276503 | A02BX | Other drugs for peptic ulcer and gastro-oesophageal reflux disease (GORD) | Other stomach medicine | _ |
| 621370301 | A02BX | Other drugs for peptic ulcer and gastro-oesophageal reflux disease (GORD) | Other stomach medicine | _ |
| 621371306 | A02BX | Other drugs for peptic ulcer and gastro-oesophageal reflux disease (GORD) | Other stomach medicine | _ |
| 621371401 | A02BX | Other drugs for peptic ulcer and gastro-oesophageal reflux disease (GORD) | Other stomach medicine | _ |
| 621575901 | A02BX | Other drugs for peptic ulcer and gastro-oesophageal reflux disease (GORD) | Other stomach medicine | _ |
| 621854102 | A02BX | Other drugs for peptic ulcer and gastro-oesophageal reflux disease (GORD) | Other stomach medicine | _ |
| 621910202 | A02BX | Other drugs for peptic ulcer and gastro-oesophageal reflux disease (GORD) | Other stomach medicine | _ |
| 621910501 | A02BX | Other drugs for peptic ulcer and gastro-oesophageal reflux disease (GORD) | Other stomach medicine | _ |
| 621915601 | A02BX | Other drugs for peptic ulcer and gastro-oesophageal reflux disease (GORD) | Other stomach medicine | _ |
| 621938401 | A02BX | Other drugs for peptic ulcer and gastro-oesophageal reflux disease (GORD) | Other stomach medicine | _ |
| 621964901 | A02BX | Other drugs for peptic ulcer and gastro-oesophageal reflux disease (GORD) | Other stomach medicine | _ |
| 621981701 | A02BX | Other drugs for peptic ulcer and gastro-oesophageal reflux disease (GORD) | Other stomach medicine | _ |
| 622009702 | A02BX | Other drugs for peptic ulcer and gastro-oesophageal reflux disease (GORD) | Other stomach medicine | _ |
| 622010901 | A02BX | Other drugs for peptic ulcer and gastro-oesophageal reflux disease (GORD) | Other stomach medicine | _ |
| 622067001 | A02BX | Other drugs for peptic ulcer and gastro-oesophageal reflux disease (GORD) | Other stomach medicine | _ |
| 622082201 | A02BX | Other drugs for peptic ulcer and gastro-oesophageal reflux disease (GORD) | Other stomach medicine | _ |
| 622525101 | A02BX | Other drugs for peptic ulcer and gastro-oesophageal reflux disease (GORD) | Other stomach medicine | _ |
| 622734200 | A02BX | Other drugs for peptic ulcer and gastro-oesophageal reflux disease (GORD) | Other stomach medicine | _ |
| 622734300 | A02BX | Other drugs for peptic ulcer and gastro-oesophageal reflux disease (GORD) | Other stomach medicine | _ |
| 622734400 | A02BX | Other drugs for peptic ulcer and gastro-oesophageal reflux disease (GORD) | Other stomach medicine | _ |
| 622734500 | A02BX | Other drugs for peptic ulcer and gastro-oesophageal reflux disease (GORD) | Other stomach medicine | _ |
| 622734600 | A02BX | Other drugs for peptic ulcer and gastro-oesophageal reflux disease (GORD) | Other stomach medicine | _ |
| 622734900 | A02BX | Other drugs for peptic ulcer and gastro-oesophageal reflux disease (GORD) | Other stomach medicine | _ |
| 622735000 | A02BX | Other drugs for peptic ulcer and gastro-oesophageal reflux disease (GORD) | Other stomach medicine | _ |
| 622735100 | A02BX | Other drugs for peptic ulcer and gastro-oesophageal reflux disease (GORD) | Other stomach medicine | _ |
| 620005988 | A02BX02 | Sucralfate | Other stomach medicine | _ |
| 620006833 | A02BX02 | Sucralfate | Other stomach medicine | _ |
| 620006834 | A02BX02 | Sucralfate | Other stomach medicine | _ |
| 620441301 | A02BX02 | Sucralfate | Other stomach medicine | _ |
| 620442002 | A02BX02 | Sucralfate | Other stomach medicine | _ |
| 620442603 | A02BX02 | Sucralfate | Other stomach medicine | _ |
| 620442701 | A02BX02 | Sucralfate | Other stomach medicine | _ |
| 622733700 | A02BX02 | Sucralfate | Other stomach medicine | _ |
| 620006085 | A02BX03 | Pirenzepine | Other stomach medicine | _ |
| 621272916 | A02BX03 | Pirenzepine | Other stomach medicine | _ |
| 622733600 | A02BX03 | Pirenzepine | Other stomach medicine | _ |
| 612320381 | A02BX11 | Troxipide | Other stomach medicine | _ |
| 620006827 | A02BX11 | Troxipide | Other stomach medicine | _ |
| 621371109 | A02BX11 | Troxipide | Other stomach medicine | _ |
| 621371211 | A02BX11 | Troxipide | Other stomach medicine | _ |
| 622734700 | A02BX11 | Troxipide | Other stomach medicine | _ |
| 622734800 | A02BX11 | Troxipide | Other stomach medicine | _ |
| 620456401 | A02BX13 | Alginic acid | Other stomach medicine | _ |
| 620456601 | A02BX13 | Alginic acid | Other stomach medicine | _ |
| 621931602 | A02BX14 | Rebamipide | Rebamipide | _ |
| 622533701 | A02BX14 | Rebamipide | Rebamipide | _ |
| 620427001 | A16AA03 | Glutamine | Other stomach medicine | _ |
| 620004435 | A16AX | Various alimentary tract and metabolism products | Other stomach medicine | _ |
| 620428301 | A16AX | Various alimentary tract and metabolism products | Other stomach medicine | _ |
| 621270401 | A16AX | Various alimentary tract and metabolism products | Other stomach medicine | _ |
| 610463227 | B01AA03 | Warfarin | Anticoagulants | _ |
| 610463228 | B01AA03 | Warfarin | Anticoagulants | _ |
| 613330001 | B01AA03 | Warfarin | Anticoagulants | _ |
| 613330002 | B01AA03 | Warfarin | Anticoagulants | _ |
| 613330003 | B01AA03 | Warfarin | Anticoagulants | _ |
| 613330004 | B01AA03 | Warfarin | Anticoagulants | _ |
| 620002332 | B01AA03 | Warfarin | Anticoagulants | _ |
| 620811502 | B01AA03 | Warfarin | Anticoagulants | _ |
| 620811503 | B01AA03 | Warfarin | Anticoagulants | _ |
| 620811507 | B01AA03 | Warfarin | Anticoagulants | _ |
| 620811510 | B01AA03 | Warfarin | Anticoagulants | _ |
| 620811511 | B01AA03 | Warfarin | Anticoagulants | _ |
| 621480504 | B01AA03 | Warfarin | Anticoagulants | _ |
| 621480506 | B01AA03 | Warfarin | Anticoagulants | _ |
| 621480507 | B01AA03 | Warfarin | Anticoagulants | _ |
| 621480604 | B01AA03 | Warfarin | Anticoagulants | _ |
| 621938101 | B01AA03 | Warfarin | Anticoagulants | _ |
| 621940901 | B01AA03 | Warfarin | Anticoagulants | _ |
| 622122601 | B01AA03 | Warfarin | Anticoagulants | _ |
| 610432021 | B01AC | Platelet aggregation inhibitors excl. heparin | Antiplatelet agent | _ |
| 613390026 | B01AC | Platelet aggregation inhibitors excl. heparin | Antiplatelet agent | _ |
| 613390027 | B01AC | Platelet aggregation inhibitors excl. heparin | Antiplatelet agent | _ |
| 620002538 | B01AC | Platelet aggregation inhibitors excl. heparin | Antiplatelet agent | _ |
| 621927101 | B01AC | Platelet aggregation inhibitors excl. heparin | Antiplatelet agent | _ |
| 621927201 | B01AC | Platelet aggregation inhibitors excl. heparin | Antiplatelet agent | _ |
| 621930601 | B01AC | Platelet aggregation inhibitors excl. heparin | Antiplatelet agent | _ |
| 621930701 | B01AC | Platelet aggregation inhibitors excl. heparin | Antiplatelet agent | _ |
| 621935102 | B01AC | Platelet aggregation inhibitors excl. heparin | Antiplatelet agent | _ |
| 621935202 | B01AC | Platelet aggregation inhibitors excl. heparin | Antiplatelet agent | _ |
| 621936701 | B01AC | Platelet aggregation inhibitors excl. heparin | Antiplatelet agent | _ |
| 621937001 | B01AC | Platelet aggregation inhibitors excl. heparin | Antiplatelet agent | _ |
| 621937701 | B01AC | Platelet aggregation inhibitors excl. heparin | Antiplatelet agent | _ |
| 621937801 | B01AC | Platelet aggregation inhibitors excl. heparin | Antiplatelet agent | _ |
| 621939201 | B01AC | Platelet aggregation inhibitors excl. heparin | Antiplatelet agent | _ |
| 621939301 | B01AC | Platelet aggregation inhibitors excl. heparin | Antiplatelet agent | _ |
| 621940701 | B01AC | Platelet aggregation inhibitors excl. heparin | Antiplatelet agent | _ |
| 621940801 | B01AC | Platelet aggregation inhibitors excl. heparin | Antiplatelet agent | _ |
| 621941901 | B01AC | Platelet aggregation inhibitors excl. heparin | Antiplatelet agent | _ |
| 621942001 | B01AC | Platelet aggregation inhibitors excl. heparin | Antiplatelet agent | _ |
| 621946101 | B01AC | Platelet aggregation inhibitors excl. heparin | Antiplatelet agent | _ |
| 621946201 | B01AC | Platelet aggregation inhibitors excl. heparin | Antiplatelet agent | _ |
| 621949501 | B01AC | Platelet aggregation inhibitors excl. heparin | Antiplatelet agent | _ |
| 621949601 | B01AC | Platelet aggregation inhibitors excl. heparin | Antiplatelet agent | _ |
| 621951401 | B01AC | Platelet aggregation inhibitors excl. heparin | Antiplatelet agent | _ |
| 621951501 | B01AC | Platelet aggregation inhibitors excl. heparin | Antiplatelet agent | _ |
| 621954802 | B01AC | Platelet aggregation inhibitors excl. heparin | Antiplatelet agent | _ |
| 621954902 | B01AC | Platelet aggregation inhibitors excl. heparin | Antiplatelet agent | _ |
| 621960801 | B01AC | Platelet aggregation inhibitors excl. heparin | Antiplatelet agent | _ |
| 621960901 | B01AC | Platelet aggregation inhibitors excl. heparin | Antiplatelet agent | _ |
| 621961001 | B01AC | Platelet aggregation inhibitors excl. heparin | Antiplatelet agent | _ |
| 621961101 | B01AC | Platelet aggregation inhibitors excl. heparin | Antiplatelet agent | _ |
| 621961201 | B01AC | Platelet aggregation inhibitors excl. heparin | Antiplatelet agent | _ |
| 621961301 | B01AC | Platelet aggregation inhibitors excl. heparin | Antiplatelet agent | _ |
| 621961401 | B01AC | Platelet aggregation inhibitors excl. heparin | Antiplatelet agent | _ |
| 621961501 | B01AC | Platelet aggregation inhibitors excl. heparin | Antiplatelet agent | _ |
| 621961601 | B01AC | Platelet aggregation inhibitors excl. heparin | Antiplatelet agent | _ |
| 621961701 | B01AC | Platelet aggregation inhibitors excl. heparin | Antiplatelet agent | _ |
| 621961801 | B01AC | Platelet aggregation inhibitors excl. heparin | Antiplatelet agent | _ |
| 621961901 | B01AC | Platelet aggregation inhibitors excl. heparin | Antiplatelet agent | _ |
| 621962001 | B01AC | Platelet aggregation inhibitors excl. heparin | Antiplatelet agent | _ |
| 621962101 | B01AC | Platelet aggregation inhibitors excl. heparin | Antiplatelet agent | _ |
| 621974001 | B01AC | Platelet aggregation inhibitors excl. heparin | Antiplatelet agent | _ |
| 621974101 | B01AC | Platelet aggregation inhibitors excl. heparin | Antiplatelet agent | _ |
| 622089801 | B01AC | Platelet aggregation inhibitors excl. heparin | Antiplatelet agent | _ |
| 622089901 | B01AC | Platelet aggregation inhibitors excl. heparin | Antiplatelet agent | _ |
| 620003468 | B01AC04 | Clopidogrel | Antiplatelet agent | _ |
| 620003469 | B01AC04 | Clopidogrel | Antiplatelet agent | _ |
| 622401801 | B01AC04 | Clopidogrel | Antiplatelet agent | _ |
| 622401901 | B01AC04 | Clopidogrel | Antiplatelet agent | _ |
| 622405801 | B01AC04 | Clopidogrel | Antiplatelet agent | _ |
| 622405901 | B01AC04 | Clopidogrel | Antiplatelet agent | _ |
| 622406101 | B01AC04 | Clopidogrel | Antiplatelet agent | _ |
| 622406201 | B01AC04 | Clopidogrel | Antiplatelet agent | _ |
| 622407701 | B01AC04 | Clopidogrel | Antiplatelet agent | _ |
| 622407801 | B01AC04 | Clopidogrel | Antiplatelet agent | _ |
| 622411301 | B01AC04 | Clopidogrel | Antiplatelet agent | _ |
| 622413301 | B01AC04 | Clopidogrel | Antiplatelet agent | _ |
| 622413401 | B01AC04 | Clopidogrel | Antiplatelet agent | _ |
| 622413501 | B01AC04 | Clopidogrel | Antiplatelet agent | _ |
| 622413601 | B01AC04 | Clopidogrel | Antiplatelet agent | _ |
| 622413701 | B01AC04 | Clopidogrel | Antiplatelet agent | _ |
| 622414401 | B01AC04 | Clopidogrel | Antiplatelet agent | _ |
| 622414501 | B01AC04 | Clopidogrel | Antiplatelet agent | _ |
| 622416201 | B01AC04 | Clopidogrel | Antiplatelet agent | _ |
| 622416301 | B01AC04 | Clopidogrel | Antiplatelet agent | _ |
| 622418201 | B01AC04 | Clopidogrel | Antiplatelet agent | _ |
| 622418301 | B01AC04 | Clopidogrel | Antiplatelet agent | _ |
| 622420101 | B01AC04 | Clopidogrel | Antiplatelet agent | _ |
| 622420201 | B01AC04 | Clopidogrel | Antiplatelet agent | _ |
| 622420301 | B01AC04 | Clopidogrel | Antiplatelet agent | _ |
| 622420502 | B01AC04 | Clopidogrel | Antiplatelet agent | _ |
| 622420602 | B01AC04 | Clopidogrel | Antiplatelet agent | _ |
| 622422201 | B01AC04 | Clopidogrel | Antiplatelet agent | _ |
| 622422301 | B01AC04 | Clopidogrel | Antiplatelet agent | _ |
| 622424101 | B01AC04 | Clopidogrel | Antiplatelet agent | _ |
| 622424201 | B01AC04 | Clopidogrel | Antiplatelet agent | _ |
| 622424301 | B01AC04 | Clopidogrel | Antiplatelet agent | _ |
| 622425001 | B01AC04 | Clopidogrel | Antiplatelet agent | _ |
| 622425101 | B01AC04 | Clopidogrel | Antiplatelet agent | _ |
| 622425501 | B01AC04 | Clopidogrel | Antiplatelet agent | _ |
| 622425601 | B01AC04 | Clopidogrel | Antiplatelet agent | _ |
| 622425701 | B01AC04 | Clopidogrel | Antiplatelet agent | _ |
| 622427501 | B01AC04 | Clopidogrel | Antiplatelet agent | _ |
| 622427601 | B01AC04 | Clopidogrel | Antiplatelet agent | _ |
| 622428401 | B01AC04 | Clopidogrel | Antiplatelet agent | _ |
| 622428501 | B01AC04 | Clopidogrel | Antiplatelet agent | _ |
| 622429501 | B01AC04 | Clopidogrel | Antiplatelet agent | _ |
| 622429601 | B01AC04 | Clopidogrel | Antiplatelet agent | _ |
| 622429701 | B01AC04 | Clopidogrel | Antiplatelet agent | _ |
| 622430501 | B01AC04 | Clopidogrel | Antiplatelet agent | _ |
| 622430601 | B01AC04 | Clopidogrel | Antiplatelet agent | _ |
| 622430701 | B01AC04 | Clopidogrel | Antiplatelet agent | _ |
| 622431201 | B01AC04 | Clopidogrel | Antiplatelet agent | _ |
| 622431301 | B01AC04 | Clopidogrel | Antiplatelet agent | _ |
| 622431501 | B01AC04 | Clopidogrel | Antiplatelet agent | _ |
| 622431601 | B01AC04 | Clopidogrel | Antiplatelet agent | _ |
| 622432801 | B01AC04 | Clopidogrel | Antiplatelet agent | _ |
| 622432901 | B01AC04 | Clopidogrel | Antiplatelet agent | _ |
| 622433001 | B01AC04 | Clopidogrel | Antiplatelet agent | _ |
| 622433101 | B01AC04 | Clopidogrel | Antiplatelet agent | _ |
| 622433201 | B01AC04 | Clopidogrel | Antiplatelet agent | _ |
| 622433301 | B01AC04 | Clopidogrel | Antiplatelet agent | _ |
| 622433401 | B01AC04 | Clopidogrel | Antiplatelet agent | _ |
| 622434501 | B01AC04 | Clopidogrel | Antiplatelet agent | _ |
| 622434601 | B01AC04 | Clopidogrel | Antiplatelet agent | _ |
| 622435501 | B01AC04 | Clopidogrel | Antiplatelet agent | _ |
| 622435601 | B01AC04 | Clopidogrel | Antiplatelet agent | _ |
| 622438201 | B01AC04 | Clopidogrel | Antiplatelet agent | _ |
| 622438301 | B01AC04 | Clopidogrel | Antiplatelet agent | _ |
| 622439501 | B01AC04 | Clopidogrel | Antiplatelet agent | _ |
| 622439601 | B01AC04 | Clopidogrel | Antiplatelet agent | _ |
| 622439801 | B01AC04 | Clopidogrel | Antiplatelet agent | _ |
| 622439901 | B01AC04 | Clopidogrel | Antiplatelet agent | _ |
| 622475400 | B01AC04 | Clopidogrel | Antiplatelet agent | _ |
| 622475500 | B01AC04 | Clopidogrel | Antiplatelet agent | _ |
| 622641701 | B01AC04 | Clopidogrel | Antiplatelet agent | _ |
| 622641801 | B01AC04 | Clopidogrel | Antiplatelet agent | _ |
| 610461073 | B01AC05 | Ticlopidine | Antiplatelet agent | _ |
| 613390007 | B01AC05 | Ticlopidine | Antiplatelet agent | _ |
| 620814301 | B01AC05 | Ticlopidine | Antiplatelet agent | _ |
| 620814502 | B01AC05 | Ticlopidine | Antiplatelet agent | _ |
| 620814505 | B01AC05 | Ticlopidine | Antiplatelet agent | _ |
| 620814506 | B01AC05 | Ticlopidine | Antiplatelet agent | _ |
| 620814511 | B01AC05 | Ticlopidine | Antiplatelet agent | _ |
| 620814512 | B01AC05 | Ticlopidine | Antiplatelet agent | _ |
| 620814521 | B01AC05 | Ticlopidine | Antiplatelet agent | _ |
| 620814531 | B01AC05 | Ticlopidine | Antiplatelet agent | _ |
| 620814543 | B01AC05 | Ticlopidine | Antiplatelet agent | _ |
| 620814601 | B01AC05 | Ticlopidine | Antiplatelet agent | _ |
| 621374002 | B01AC05 | Ticlopidine | Antiplatelet agent | _ |
| 622738700 | B01AC05 | Ticlopidine | Antiplatelet agent | _ |
| 610443053 | B01AC06 | Acetylsalicylic acid | Antiplatelet agent | _ |
| 620007816 | B01AC06 | Acetylsalicylic acid | Antiplatelet agent | _ |
| 621525202 | B01AC06 | Acetylsalicylic acid | Antiplatelet agent | _ |
| 621675501 | B01AC06 | Acetylsalicylic acid | Antiplatelet agent | _ |
| 621676502 | B01AC06 | Acetylsalicylic acid | Antiplatelet agent | _ |
| 622258001 | B01AC06 | Acetylsalicylic acid | Antiplatelet agent | _ |
| 613390023 | B01AC19 | Beraprost | Antiplatelet agent | _ |
| 613390024 | B01AC19 | Beraprost | Antiplatelet agent | _ |
| 621481301 | B01AC19 | Beraprost | Antiplatelet agent | _ |
| 621481401 | B01AC19 | Beraprost | Antiplatelet agent | _ |
| 621481404 | B01AC19 | Beraprost | Antiplatelet agent | _ |
| 621481501 | B01AC19 | Beraprost | Antiplatelet agent | _ |
| 621481601 | B01AC19 | Beraprost | Antiplatelet agent | _ |
| 621481801 | B01AC19 | Beraprost | Antiplatelet agent | _ |
| 621481904 | B01AC19 | Beraprost | Antiplatelet agent | _ |
| 621482001 | B01AC19 | Beraprost | Antiplatelet agent | _ |
| 621482101 | B01AC19 | Beraprost | Antiplatelet agent | _ |
| 621482302 | B01AC19 | Beraprost | Antiplatelet agent | _ |
| 621526401 | B01AC19 | Beraprost | Antiplatelet agent | _ |
| 621530401 | B01AC19 | Beraprost | Antiplatelet agent | _ |
| 621639901 | B01AC19 | Beraprost | Antiplatelet agent | _ |
| 621640001 | B01AC19 | Beraprost | Antiplatelet agent | _ |
| 622147201 | B01AC19 | Beraprost | Antiplatelet agent | _ |
| 622739200 | B01AC19 | Beraprost | Antiplatelet agent | _ |
| 622739300 | B01AC19 | Beraprost | Antiplatelet agent | _ |
| 622336601 | B01AC22 | Prasugrel | Antiplatelet agent | _ |
| 622336701 | B01AC22 | Prasugrel | Antiplatelet agent | _ |
| 622452301 | B01AC22 | Prasugrel | Antiplatelet agent | _ |
| 622658901 | B01AC22 | Prasugrel | Antiplatelet agent | _ |
| 610444056 | B01AC23 | Cilostazol | Antiplatelet agent | _ |
| 610444140 | B01AC23 | Cilostazol | Antiplatelet agent | _ |
| 610444141 | B01AC23 | Cilostazol | Antiplatelet agent | _ |
| 620003450 | B01AC23 | Cilostazol | Antiplatelet agent | _ |
| 620003451 | B01AC23 | Cilostazol | Antiplatelet agent | _ |
| 620005094 | B01AC23 | Cilostazol | Antiplatelet agent | _ |
| 620009320 | B01AC23 | Cilostazol | Antiplatelet agent | _ |
| 620009321 | B01AC23 | Cilostazol | Antiplatelet agent | _ |
| 621312902 | B01AC23 | Cilostazol | Antiplatelet agent | _ |
| 621313101 | B01AC23 | Cilostazol | Antiplatelet agent | _ |
| 621313301 | B01AC23 | Cilostazol | Antiplatelet agent | _ |
| 621313502 | B01AC23 | Cilostazol | Antiplatelet agent | _ |
| 621313601 | B01AC23 | Cilostazol | Antiplatelet agent | _ |
| 621313702 | B01AC23 | Cilostazol | Antiplatelet agent | _ |
| 621313801 | B01AC23 | Cilostazol | Antiplatelet agent | _ |
| 621314101 | B01AC23 | Cilostazol | Antiplatelet agent | _ |
| 621314301 | B01AC23 | Cilostazol | Antiplatelet agent | _ |
| 621314601 | B01AC23 | Cilostazol | Antiplatelet agent | _ |
| 621314802 | B01AC23 | Cilostazol | Antiplatelet agent | _ |
| 621314901 | B01AC23 | Cilostazol | Antiplatelet agent | _ |
| 621315002 | B01AC23 | Cilostazol | Antiplatelet agent | _ |
| 621315201 | B01AC23 | Cilostazol | Antiplatelet agent | _ |
| 621418301 | B01AC23 | Cilostazol | Antiplatelet agent | _ |
| 621418401 | B01AC23 | Cilostazol | Antiplatelet agent | _ |
| 621418501 | B01AC23 | Cilostazol | Antiplatelet agent | _ |
| 621418601 | B01AC23 | Cilostazol | Antiplatelet agent | _ |
| 621418701 | B01AC23 | Cilostazol | Antiplatelet agent | _ |
| 621418802 | B01AC23 | Cilostazol | Antiplatelet agent | _ |
| 621523902 | B01AC23 | Cilostazol | Antiplatelet agent | _ |
| 621524002 | B01AC23 | Cilostazol | Antiplatelet agent | _ |
| 621638701 | B01AC23 | Cilostazol | Antiplatelet agent | _ |
| 621721201 | B01AC23 | Cilostazol | Antiplatelet agent | _ |
| 621721301 | B01AC23 | Cilostazol | Antiplatelet agent | _ |
| 621971201 | B01AC23 | Cilostazol | Antiplatelet agent | _ |
| 621971301 | B01AC23 | Cilostazol | Antiplatelet agent | _ |
| 622226901 | B01AC23 | Cilostazol | Antiplatelet agent | _ |
| 622227001 | B01AC23 | Cilostazol | Antiplatelet agent | _ |
| 622256201 | B01AC23 | Cilostazol | Antiplatelet agent | _ |
| 622256301 | B01AC23 | Cilostazol | Antiplatelet agent | _ |
| 622315200 | B01AC23 | Cilostazol | Antiplatelet agent | _ |
| 622315300 | B01AC23 | Cilostazol | Antiplatelet agent | _ |
| 622334101 | B01AC23 | Cilostazol | Antiplatelet agent | _ |
| 622334201 | B01AC23 | Cilostazol | Antiplatelet agent | _ |
| 622344001 | B01AC23 | Cilostazol | Antiplatelet agent | _ |
| 622344101 | B01AC23 | Cilostazol | Antiplatelet agent | _ |
| 622346301 | B01AC23 | Cilostazol | Antiplatelet agent | _ |
| 622347901 | B01AC23 | Cilostazol | Antiplatelet agent | _ |
| 622361301 | B01AC23 | Cilostazol | Antiplatelet agent | _ |
| 622373001 | B01AC23 | Cilostazol | Antiplatelet agent | _ |
| 622373101 | B01AC23 | Cilostazol | Antiplatelet agent | _ |
| 622384101 | B01AC23 | Cilostazol | Antiplatelet agent | _ |
| 622384201 | B01AC23 | Cilostazol | Antiplatelet agent | _ |
| 622501801 | B01AC23 | Cilostazol | Antiplatelet agent | _ |
| 622501901 | B01AC23 | Cilostazol | Antiplatelet agent | _ |
| 622472201 | B01AC24 | Ticagrelor | Antiplatelet agent | _ |
| 622472301 | B01AC24 | Ticagrelor | Antiplatelet agent | _ |
| 621374801 | B01AC30 | Combinations | Antiplatelet agent | _ |
| 621374901 | B01AC30 | Combinations | Antiplatelet agent | _ |
| 621375001 | B01AC30 | Combinations | Antiplatelet agent | _ |
| 621419201 | B01AC30 | Combinations | Antiplatelet agent | _ |
| 621419401 | B01AC30 | Combinations | Antiplatelet agent | _ |
| 622290301 | B01AC30 | Combinations | Antiplatelet agent | _ |
| 622828901 | B01AC30 | Combinations | Antiplatelet agent | _ |
| 622829701 | B01AC30 | Combinations | Antiplatelet agent | _ |
| 622833001 | B01AC30 | Combinations | Antiplatelet agent | _ |
| 622341601 | B01AC56 | Acetylsalicylic acid, combinations with proton pump inhibitors | Antiplatelet agent | _ |
| 622795301 | B01AC56 | Acetylsalicylic acid, combinations with proton pump inhibitors | Antiplatelet agent | _ |
| 622043301 | B01AE07 | Dabigatran etexilate | Anticoagulants | _ |
| 622043401 | B01AE07 | Dabigatran etexilate | Anticoagulants | _ |
| 622080901 | B01AF | Direct factor Xa inhibitors | Anticoagulants | _ |
| 622081001 | B01AF | Direct factor Xa inhibitors | Anticoagulants | _ |
| 622375201 | B01AF | Direct factor Xa inhibitors | Anticoagulants | _ |
| 622068301 | B01AF01 | Rivaroxaban | Anticoagulants | _ |
| 622068401 | B01AF01 | Rivaroxaban | Anticoagulants | _ |
| 622449101 | B01AF01 | Rivaroxaban | Anticoagulants | _ |
| 622449201 | B01AF01 | Rivaroxaban | Anticoagulants | _ |
| 622829001 | B01AF01 | Rivaroxaban | Anticoagulants | _ |
| 622829101 | B01AF01 | Rivaroxaban | Anticoagulants | _ |
| 622853901 | B01AF01 | Rivaroxaban | Anticoagulants | _ |
| 622854001 | B01AF01 | Rivaroxaban | Anticoagulants | _ |
| 622224901 | B01AF02 | Apixaban | Anticoagulants | _ |
| 622225001 | B01AF02 | Apixaban | Anticoagulants | _ |
| 622576001 | B01AF03 | Edoxaban | Anticoagulants | _ |
| 622576101 | B01AF03 | Edoxaban | Anticoagulants | _ |
| 622576201 | B01AF03 | Edoxaban | Anticoagulants | _ |
| 622462301 | B02BX | Other systemic hemostatics | Antiplatelet agent | _ |
| 621512701 | B05BB01 | Electrolytes | Antiplatelet agent | _ |
| 610421343 | C10AX06 | Omega-3-triglycerides incl. Other esters and acids | Antiplatelet agent | _ |
| 610421344 | C10AX06 | Omega-3-triglycerides incl. Other esters and acids | Antiplatelet agent | _ |
| 610422056 | C10AX06 | Omega-3-triglycerides incl. Other esters and acids | Antiplatelet agent | _ |
| 613390006 | C10AX06 | Omega-3-triglycerides incl. Other esters and acids | Antiplatelet agent | _ |
| 620001924 | C10AX06 | Omega-3-triglycerides incl. Other esters and acids | Antiplatelet agent | _ |
| 620005932 | C10AX06 | Omega-3-triglycerides incl. Other esters and acids | Antiplatelet agent | _ |
| 620007887 | C10AX06 | Omega-3-triglycerides incl. Other esters and acids | Antiplatelet agent | _ |
| 620007888 | C10AX06 | Omega-3-triglycerides incl. Other esters and acids | Antiplatelet agent | _ |
| 620007889 | C10AX06 | Omega-3-triglycerides incl. Other esters and acids | Antiplatelet agent | _ |
| 620007890 | C10AX06 | Omega-3-triglycerides incl. Other esters and acids | Antiplatelet agent | _ |
| 620007891 | C10AX06 | Omega-3-triglycerides incl. Other esters and acids | Antiplatelet agent | _ |
| 620007892 | C10AX06 | Omega-3-triglycerides incl. Other esters and acids | Antiplatelet agent | _ |
| 620007893 | C10AX06 | Omega-3-triglycerides incl. Other esters and acids | Antiplatelet agent | _ |
| 620007894 | C10AX06 | Omega-3-triglycerides incl. Other esters and acids | Antiplatelet agent | _ |
| 620007895 | C10AX06 | Omega-3-triglycerides incl. Other esters and acids | Antiplatelet agent | _ |
| 620007896 | C10AX06 | Omega-3-triglycerides incl. Other esters and acids | Antiplatelet agent | _ |
| 620007897 | C10AX06 | Omega-3-triglycerides incl. Other esters and acids | Antiplatelet agent | _ |
| 620007898 | C10AX06 | Omega-3-triglycerides incl. Other esters and acids | Antiplatelet agent | _ |
| 620007993 | C10AX06 | Omega-3-triglycerides incl. Other esters and acids | Antiplatelet agent | _ |
| 620007994 | C10AX06 | Omega-3-triglycerides incl. Other esters and acids | Antiplatelet agent | _ |
| 620007995 | C10AX06 | Omega-3-triglycerides incl. Other esters and acids | Antiplatelet agent | _ |
| 620008112 | C10AX06 | Omega-3-triglycerides incl. Other esters and acids | Antiplatelet agent | _ |
| 620008113 | C10AX06 | Omega-3-triglycerides incl. Other esters and acids | Antiplatelet agent | _ |
| 620815302 | C10AX06 | Omega-3-triglycerides incl. other esters and acids | Antiplatelet agent | _ |
| 620815601 | C10AX06 | Omega-3-triglycerides incl. Other esters and acids | Antiplatelet agent | _ |
| 620815903 | C10AX06 | Omega-3-triglycerides incl. Other esters and acids | Antiplatelet agent | _ |
| 620816201 | C10AX06 | Omega-3-triglycerides incl. Other esters and acids | Antiplatelet agent | _ |
| 620816301 | C10AX06 | Omega-3-triglycerides incl. other esters and acids | Antiplatelet agent | _ |
| 620816501 | C10AX06 | Omega-3-triglycerides incl. Other esters and acids | Antiplatelet agent | _ |
| 620817001 | C10AX06 | Omega-3-triglycerides incl. other esters and acids | Antiplatelet agent | _ |
| 620817103 | C10AX06 | Omega-3-triglycerides incl. other esters and acids | Antiplatelet agent | _ |
| 620817504 | C10AX06 | Omega-3-triglycerides incl. Other esters and acids | Antiplatelet agent | _ |
| 620817702 | C10AX06 | Omega-3-triglycerides incl. Other esters and acids | Antiplatelet agent | _ |
| 621315603 | C10AX06 | Omega-3-triglycerides incl. other esters and acids | Antiplatelet agent | _ |
| 621776502 | C10AX06 | Omega-3-triglycerides incl. other esters and acids | Antiplatelet agent | _ |
| 621869505 | C10AX06 | Omega-3-triglycerides incl. other esters and acids | Antiplatelet agent | _ |
| 621869605 | C10AX06 | Omega-3-triglycerides incl. other esters and acids | Antiplatelet agent | _ |
| 621869705 | C10AX06 | Omega-3-triglycerides incl. other esters and acids | Antiplatelet agent | _ |
| 621959902 | C10AX06 | Omega-3-triglycerides incl. other esters and acids | Antiplatelet agent | _ |
| 621960002 | C10AX06 | Omega-3-triglycerides incl. other esters and acids | Antiplatelet agent | _ |
| 621960102 | C10AX06 | Omega-3-triglycerides incl. other esters and acids | Antiplatelet agent | _ |
| 622468501 | C10AX06 | Omega-3-triglycerides incl. Other esters and acids | Antiplatelet agent | _ |
| 622468601 | C10AX06 | Omega-3-triglycerides incl. Other esters and acids | Antiplatelet agent | _ |
| 622468701 | C10AX06 | Omega-3-triglycerides incl. Other esters and acids | Antiplatelet agent | _ |
| 622610500 | C10AX06 | Omega-3-triglycerides incl. other esters and acids | Antiplatelet agent | _ |
| 622738900 | C10AX06 | Omega-3-triglycerides incl. other esters and acids | Antiplatelet agent | _ |
| 622739000 | C10AX06 | Omega-3-triglycerides incl. other esters and acids | Antiplatelet agent | _ |
| 622739100 | C10AX06 | Omega-3-triglycerides incl. other esters and acids | Antiplatelet agent | _ |
| 620007078 | H02AA02 | Fludrocortisone | Steroid | _ |
| 620004578 | H02AB01 | Betamethasone | Steroid | _ |
| 620004620 | H02AB01 | Betamethasone | Steroid | _ |
| 620005133 | H02AB01 | Betamethasone | Steroid | _ |
| 620005134 | H02AB01 | Betamethasone | Steroid | _ |
| 620521501 | H02AB01 | Betamethasone | Steroid | _ |
| 620006985 | H02AB02 | Dexamethasone | Steroid | _ |
| 620006986 | H02AB02 | Dexamethasone | Steroid | _ |
| 620521302 | H02AB02 | Dexamethasone | Steroid | _ |
| 621997701 | H02AB02 | Dexamethasone | Steroid | _ |
| 622359901 | H02AB02 | Dexamethasone | Steroid | _ |
| 620005125 | H02AB04 | Methylprednisolone | Steroid | _ |
| 620005126 | H02AB04 | Methylprednisolone | Steroid | _ |
| 610422253 | H02AB06 | Prednisolone | Steroid | _ |
| 610431117 | H02AB06 | Prednisolone | Steroid | _ |
| 610454071 | H02AB06 | Prednisolone | Steroid | _ |
| 612450051 | H02AB06 | Prednisolone | Steroid | _ |
| 612450096 | H02AB06 | Prednisolone | Steroid | _ |
| 612450118 | H02AB06 | Prednisolone | Steroid | _ |
| 620000125 | H02AB06 | Prednisolone | Steroid | _ |
| 620000694 | H02AB06 | Prednisolone | Steroid | _ |
| 620000695 | H02AB06 | Prednisolone | Steroid | _ |
| 620000696 | H02AB06 | Prednisolone | Steroid | _ |
| 620004294 | H02AB06 | Prednisolone | Steroid | _ |
| 620004387 | H02AB06 | Prednisolone | Steroid | _ |
| 620005848 | H02AB06 | Prednisolone | Steroid | _ |
| 620527133 | H02AB06 | Prednisolone | Steroid | _ |
| 620527205 | H02AB06 | Prednisolone | Steroid | _ |
| 621559301 | H02AB06 | Prednisolone | Steroid | _ |
| 642450169 | H02AB06 | Prednisolone | Steroid | _ |
| 642450170 | H02AB06 | Prednisolone | Steroid | _ |
| 642450171 | H02AB06 | Prednisolone | Steroid | _ |
| 612450070 | H02AB08 | Triamcinolone | Steroid | _ |
| 620006903 | H02AB09 | Hydrocortisone | Steroid | _ |
| 620002513 | H02AB10 | Cortisone | Steroid | _ |
| 620530701 | H02BX | Corticosteroids for systemic use, combinations | Steroid | _ |
| 620530801 | H02BX | Corticosteroids for systemic use, combinations | Steroid | _ |
| 620531001 | H02BX | Corticosteroids for systemic use, combinations | Steroid | _ |
| 620531101 | H02BX | Corticosteroids for systemic use, combinations | Steroid | _ |
| 620531301 | H02BX | Corticosteroids for systemic use, combinations | Steroid | _ |
| 620531401 | H02BX | Corticosteroids for systemic use, combinations | Steroid | _ |
| 620531501 | H02BX | Corticosteroids for systemic use, combinations | Steroid | _ |
| 620531601 | H02BX | Corticosteroids for systemic use, combinations | Steroid | _ |
| 620007095 | M01AB05 | Diclofenac | Diclofenac | 37.5 |
| 620007096 | M01AB05 | Diclofenac | Diclofenac | 25 |
| 620008628 | M01AB05 | Diclofenac | Diclofenac | 37.5 |
| 620079303 | M01AB05 | Diclofenac | Diclofenac | 25 |
| 620079305 | M01AB05 | Diclofenac | Diclofenac | 25 |
| 620079311 | M01AB05 | Diclofenac | Diclofenac | 25 |
| 620079315 | M01AB05 | Diclofenac | Diclofenac | 25 |
| 620079325 | M01AB05 | Diclofenac | Diclofenac | 25 |
| 620079338 | M01AB05 | Diclofenac | Diclofenac | 25 |
| 620079345 | M01AB05 | Diclofenac | Diclofenac | 25 |
| 620079349 | M01AB05 | Diclofenac | Diclofenac | 25 |
| 620080001 | M01AB05 | Diclofenac | Diclofenac | 37.5 |
| 621212601 | M01AB05 | Diclofenac | Diclofenac | 37.5 |
| 622709400 | M01AB05 | Diclofenac | Diclofenac | 25 |
| 622709500 | M01AB05 | Diclofenac | Diclofenac | 37.5 |
| 622770400 | M01AB05 | Diclofenac | Diclofenac | 25 |
| 622770500 | M01AB05 | Diclofenac | Diclofenac | 50 |
| 620002431 | M01AC06 | Meloxicam | Meloxicam | 5 |
| 620002432 | M01AC06 | Meloxicam | Meloxicam | 10 |
| 620008114 | M01AC06 | Meloxicam | Meloxicam | 5 |
| 620008115 | M01AC06 | Meloxicam | Meloxicam | 5 |
| 620008117 | M01AC06 | Meloxicam | Meloxicam | 5 |
| 620008118 | M01AC06 | Meloxicam | Meloxicam | 5 |
| 620008119 | M01AC06 | Meloxicam | Meloxicam | 5 |
| 620008120 | M01AC06 | Meloxicam | Meloxicam | 5 |
| 620008121 | M01AC06 | Meloxicam | Meloxicam | 5 |
| 620008124 | M01AC06 | Meloxicam | Meloxicam | 5 |
| 620008125 | M01AC06 | Meloxicam | Meloxicam | 5 |
| 620008127 | M01AC06 | Meloxicam | Meloxicam | 5 |
| 620008128 | M01AC06 | Meloxicam | Meloxicam | 5 |
| 620008129 | M01AC06 | Meloxicam | Meloxicam | 5 |
| 620008131 | M01AC06 | Meloxicam | Meloxicam | 5 |
| 620008132 | M01AC06 | Meloxicam | Meloxicam | 10 |
| 620008133 | M01AC06 | Meloxicam | Meloxicam | 10 |
| 620008135 | M01AC06 | Meloxicam | Meloxicam | 10 |
| 620008136 | M01AC06 | Meloxicam | Meloxicam | 10 |
| 620008137 | M01AC06 | Meloxicam | Meloxicam | 10 |
| 620008138 | M01AC06 | Meloxicam | Meloxicam | 10 |
| 620008139 | M01AC06 | Meloxicam | Meloxicam | 10 |
| 620008142 | M01AC06 | Meloxicam | Meloxicam | 10 |
| 620008143 | M01AC06 | Meloxicam | Meloxicam | 10 |
| 620008145 | M01AC06 | Meloxicam | Meloxicam | 10 |
| 620008146 | M01AC06 | Meloxicam | Meloxicam | 10 |
| 620008147 | M01AC06 | Meloxicam | Meloxicam | 10 |
| 620008149 | M01AC06 | Meloxicam | Meloxicam | 10 |
| 620008150 | M01AC06 | Meloxicam | Meloxicam | 5 |
| 620008151 | M01AC06 | Meloxicam | Meloxicam | 10 |
| 621837703 | M01AC06 | Meloxicam | Meloxicam | 5 |
| 621837803 | M01AC06 | Meloxicam | Meloxicam | 10 |
| 620007150 | M01AE | Propionic acid derivatives | Loxoprofen | 60 |
| 620007151 | M01AE | Propionic acid derivatives | Loxoprofen | 60 |
| 620007152 | M01AE | Propionic acid derivatives | Loxoprofen | 60 |
| 620098401 | M01AE | Propionic acid derivatives | Loxoprofen | 60 |
| 620098501 | M01AE | Propionic acid derivatives | Loxoprofen | 60 |
| 620098702 | M01AE | Propionic acid derivatives | Loxoprofen | 60 |
| 620098801 | M01AE | Propionic acid derivatives | Loxoprofen | 60 |
| 620098902 | M01AE | Propionic acid derivatives | Loxoprofen | 60 |
| 620099003 | M01AE | Propionic acid derivatives | Loxoprofen | 60 |
| 620099101 | M01AE | Propionic acid derivatives | Loxoprofen | 60 |
| 620099201 | M01AE | Propionic acid derivatives | Loxoprofen | 60 |
| 620099301 | M01AE | Propionic acid derivatives | Loxoprofen | 60 |
| 620099501 | M01AE | Propionic acid derivatives | Loxoprofen | 60 |
| 620099602 | M01AE | Propionic acid derivatives | Loxoprofen | 60 |
| 620099701 | M01AE | Propionic acid derivatives | Loxoprofen | 60 |
| 620100001 | M01AE | Propionic acid derivatives | Loxoprofen | 60 |
| 620100401 | M01AE | Propionic acid derivatives | Loxoprofen | 60 |
| 620100501 | M01AE | Propionic acid derivatives | Loxoprofen | 60 |
| 620100602 | M01AE | Propionic acid derivatives | Loxoprofen | 60 |
| 620100702 | M01AE | Propionic acid derivatives | Loxoprofen | 60 |
| 620100901 | M01AE | Propionic acid derivatives | Loxoprofen | 60 |
| 621215101 | M01AE | Propionic acid derivatives | Loxoprofen | 60 |
| 621215301 | M01AE | Propionic acid derivatives | Loxoprofen | 60 |
| 621215401 | M01AE | Propionic acid derivatives | Loxoprofen | 60 |
| 621215602 | M01AE | Propionic acid derivatives | Loxoprofen | 60 |
| 621392002 | M01AE | Propionic acid derivatives | Loxoprofen | 60 |
| 621534501 | M01AE | Propionic acid derivatives | Loxoprofen | 60 |
| 621808201 | M01AE | Propionic acid derivatives | Loxoprofen | 60 |
| 622325600 | M01AE | Propionic acid derivatives | Loxoprofen | 60 |
| 622710200 | M01AE | Propionic acid derivatives | Loxoprofen | 60 |
| 620006849 | M01AE01 | Ibuprofen | Ibuprofen | 100 |
| 620007068 | M01AE01 | Ibuprofen | Ibuprofen | 200 |
| 620088902 | M01AE01 | Ibuprofen | Ibuprofen | 200 |
| 620089301 | M01AE01 | Ibuprofen | Ibuprofen | 100 |
| 620090601 | M01AE01 | Ibuprofen | Ibuprofen | 100 |
| 622058201 | M01AE01 | Ibuprofen | Ibuprofen | 200 |
| 622066701 | M01AE01 | Ibuprofen | Ibuprofen | 200 |
| 622066702 | M01AE01 | Ibuprofen | Ibuprofen | 200 |
| 622709600 | M01AE01 | Ibuprofen | Ibuprofen | 200 |
| 622709700 | M01AE01 | Ibuprofen | Ibuprofen | 100 |
| 622709800 | M01AE01 | Ibuprofen | Ibuprofen | 200 |
| 620004857 | M01AH01 | Celecoxib | Celecoxib | 100 |
| 620004858 | M01AH01 | Celecoxib | Celecoxib | 200 |
| 622704201 | M01AH01 | Celecoxib | Celecoxib | 100 |
| 622704301 | M01AH01 | Celecoxib | Celecoxib | 200 |
| 622783201 | M01AH01 | Celecoxib | Celecoxib | 100 |
| 622783301 | M01AH01 | Celecoxib | Celecoxib | 200 |
| 622785001 | M01AH01 | Celecoxib | Celecoxib | 100 |
| 622785101 | M01AH01 | Celecoxib | Celecoxib | 200 |
| 622786401 | M01AH01 | Celecoxib | Celecoxib | 100 |
| 622786501 | M01AH01 | Celecoxib | Celecoxib | 200 |
| 622787601 | M01AH01 | Celecoxib | Celecoxib | 100 |
| 622787701 | M01AH01 | Celecoxib | Celecoxib | 200 |
| 622788301 | M01AH01 | Celecoxib | Celecoxib | 100 |
| 622788401 | M01AH01 | Celecoxib | Celecoxib | 200 |
| 622795401 | M01AH01 | Celecoxib | Celecoxib | 100 |
| 622795501 | M01AH01 | Celecoxib | Celecoxib | 200 |
| 622797301 | M01AH01 | Celecoxib | Celecoxib | 100 |
| 622797401 | M01AH01 | Celecoxib | Celecoxib | 200 |
| 622797801 | M01AH01 | Celecoxib | Celecoxib | 100 |
| 622797901 | M01AH01 | Celecoxib | Celecoxib | 200 |
| 622799301 | M01AH01 | Celecoxib | Celecoxib | 100 |
| 622799401 | M01AH01 | Celecoxib | Celecoxib | 200 |
| 622799701 | M01AH01 | Celecoxib | Celecoxib | 100 |
| 622799801 | M01AH01 | Celecoxib | Celecoxib | 200 |
| 622801301 | M01AH01 | Celecoxib | Celecoxib | 100 |
| 622801401 | M01AH01 | Celecoxib | Celecoxib | 200 |
| 622803801 | M01AH01 | Celecoxib | Celecoxib | 100 |
| 622803901 | M01AH01 | Celecoxib | Celecoxib | 200 |
| 622805401 | M01AH01 | Celecoxib | Celecoxib | 100 |
| 622805501 | M01AH01 | Celecoxib | Celecoxib | 200 |
| 622806501 | M01AH01 | Celecoxib | Celecoxib | 100 |
| 622806601 | M01AH01 | Celecoxib | Celecoxib | 200 |
| 622808901 | M01AH01 | Celecoxib | Celecoxib | 100 |
| 622809001 | M01AH01 | Celecoxib | Celecoxib | 200 |
| 622809101 | M01AH01 | Celecoxib | Celecoxib | 100 |
| 622809201 | M01AH01 | Celecoxib | Celecoxib | 200 |
| 622811301 | M01AH01 | Celecoxib | Celecoxib | 100 |
| 622811401 | M01AH01 | Celecoxib | Celecoxib | 200 |
| 622845300 | M01AH01 | Celecoxib | Celecoxib | 100 |
| 622845400 | M01AH01 | Celecoxib | Celecoxib | 200 |
| 620009099 | M05BA | Bisphosphonates | Bisphosphonate | _ |
| 620009100 | M05BA | Bisphosphonates | Bisphosphonate | _ |
| 622093101 | M05BA | Bisphosphonates | Bisphosphonate | _ |
| 622094601 | M05BA | Bisphosphonates | Bisphosphonate | _ |
| 622621101 | M05BA | Bisphosphonates | Bisphosphonate | _ |
| 622621201 | M05BA | Bisphosphonates | Bisphosphonate | _ |
| 622627301 | M05BA | Bisphosphonates | Bisphosphonate | _ |
| 622627401 | M05BA | Bisphosphonates | Bisphosphonate | _ |
| 622630301 | M05BA | Bisphosphonates | Bisphosphonate | _ |
| 622630401 | M05BA | Bisphosphonates | Bisphosphonate | _ |
| 622634301 | M05BA | Bisphosphonates | Bisphosphonate | _ |
| 622634401 | M05BA | Bisphosphonates | Bisphosphonate | _ |
| 622635701 | M05BA | Bisphosphonates | Bisphosphonate | _ |
| 622635801 | M05BA | Bisphosphonates | Bisphosphonate | _ |
| 622637301 | M05BA | Bisphosphonates | Bisphosphonate | _ |
| 622637401 | M05BA | Bisphosphonates | Bisphosphonate | _ |
| 622639001 | M05BA | Bisphosphonates | Bisphosphonate | _ |
| 622639101 | M05BA | Bisphosphonates | Bisphosphonate | _ |
| 622640401 | M05BA | Bisphosphonates | Bisphosphonate | _ |
| 622640501 | M05BA | Bisphosphonates | Bisphosphonate | _ |
| 622643701 | M05BA | Bisphosphonates | Bisphosphonate | _ |
| 622643801 | M05BA | Bisphosphonates | Bisphosphonate | _ |
| 613990084 | M05BA01 | Etidronic acid | Bisphosphonate | _ |
| 610451021 | M05BA04 | Alendronic acid | Bisphosphonate | _ |
| 620004357 | M05BA04 | Alendronic acid | Bisphosphonate | _ |
| 620004359 | M05BA04 | Alendronic acid | Bisphosphonate | _ |
| 620009303 | M05BA04 | Alendronic acid | Bisphosphonate | _ |
| 620009305 | M05BA04 | Alendronic acid | Bisphosphonate | _ |
| 621896801 | M05BA04 | Alendronic acid | Bisphosphonate | _ |
| 621977502 | M05BA04 | Alendronic acid | Bisphosphonate | _ |
| 622091301 | M05BA04 | Alendronic acid | Bisphosphonate | _ |
| 622091401 | M05BA04 | Alendronic acid | Bisphosphonate | _ |
| 622101602 | M05BA04 | Alendronic acid | Bisphosphonate | _ |
| 622110601 | M05BA04 | Alendronic acid | Bisphosphonate | _ |
| 622110701 | M05BA04 | Alendronic acid | Bisphosphonate | _ |
| 622113503 | M05BA04 | Alendronic acid | Bisphosphonate | _ |
| 622118301 | M05BA04 | Alendronic acid | Bisphosphonate | _ |
| 622121501 | M05BA04 | Alendronic acid | Bisphosphonate | _ |
| 622121601 | M05BA04 | Alendronic acid | Bisphosphonate | _ |
| 622122501 | M05BA04 | Alendronic acid | Bisphosphonate | _ |
| 622127901 | M05BA04 | Alendronic acid | Bisphosphonate | _ |
| 622128001 | M05BA04 | Alendronic acid | Bisphosphonate | _ |
| 622186801 | M05BA04 | Alendronic acid | Bisphosphonate | _ |
| 622186901 | M05BA04 | Alendronic acid | Bisphosphonate | _ |
| 622222101 | M05BA04 | Alendronic acid | Bisphosphonate | _ |
| 622222201 | M05BA04 | Alendronic acid | Bisphosphonate | _ |
| 622223501 | M05BA04 | Alendronic acid | Bisphosphonate | _ |
| 622255801 | M05BA04 | Alendronic acid | Bisphosphonate | _ |
| 622255901 | M05BA04 | Alendronic acid | Bisphosphonate | _ |
| 622291001 | M05BA04 | Alendronic acid | Bisphosphonate | _ |
| 622291101 | M05BA04 | Alendronic acid | Bisphosphonate | _ |
| 622532001 | M05BA04 | Alendronic acid | Bisphosphonate | _ |
| 622532101 | M05BA04 | Alendronic acid | Bisphosphonate | _ |
| 622760100 | M05BA04 | Alendronic acid | Bisphosphonate | _ |
| 622847200 | M05BA04 | Alendronic acid | Bisphosphonate | _ |
| 622471501 | M05BA06 | Ibandronic acid | Bisphosphonate | _ |
| 610462001 | M05BA07 | Risedronic acid | Bisphosphonate | _ |
| 610462003 | M05BA07 | Risedronic acid | Bisphosphonate | _ |
| 620004865 | M05BA07 | Risedronic acid | Bisphosphonate | _ |
| 620004871 | M05BA07 | Risedronic acid | Bisphosphonate | _ |
| 622041601 | M05BA07 | Risedronic acid | Bisphosphonate | _ |
| 622045601 | M05BA07 | Risedronic acid | Bisphosphonate | _ |
| 622050601 | M05BA07 | Risedronic acid | Bisphosphonate | _ |
| 622053501 | M05BA07 | Risedronic acid | Bisphosphonate | _ |
| 622053701 | M05BA07 | Risedronic acid | Bisphosphonate | _ |
| 622058101 | M05BA07 | Risedronic acid | Bisphosphonate | _ |
| 622061801 | M05BA07 | Risedronic acid | Bisphosphonate | _ |
| 622063401 | M05BA07 | Risedronic acid | Bisphosphonate | _ |
| 622110801 | M05BA07 | Risedronic acid | Bisphosphonate | _ |
| 622118001 | M05BA07 | Risedronic acid | Bisphosphonate | _ |
| 622122401 | M05BA07 | Risedronic acid | Bisphosphonate | _ |
| 622124401 | M05BA07 | Risedronic acid | Bisphosphonate | _ |
| 622130101 | M05BA07 | Risedronic acid | Bisphosphonate | _ |
| 622147701 | M05BA07 | Risedronic acid | Bisphosphonate | _ |
| 622159101 | M05BA07 | Risedronic acid | Bisphosphonate | _ |
| 622188801 | M05BA07 | Risedronic acid | Bisphosphonate | _ |
| 622205001 | M05BA07 | Risedronic acid | Bisphosphonate | _ |
| 622206401 | M05BA07 | Risedronic acid | Bisphosphonate | _ |
| 622219901 | M05BA07 | Risedronic acid | Bisphosphonate | _ |
| 622224701 | M05BA07 | Risedronic acid | Bisphosphonate | _ |
| 622226001 | M05BA07 | Risedronic acid | Bisphosphonate | _ |
| 622227101 | M05BA07 | Risedronic acid | Bisphosphonate | _ |
| 622228901 | M05BA07 | Risedronic acid | Bisphosphonate | _ |
| 622229301 | M05BA07 | Risedronic acid | Bisphosphonate | _ |
| 622233301 | M05BA07 | Risedronic acid | Bisphosphonate | _ |
| 622235101 | M05BA07 | Risedronic acid | Bisphosphonate | _ |
| 622235201 | M05BA07 | Risedronic acid | Bisphosphonate | _ |
| 622236401 | M05BA07 | Risedronic acid | Bisphosphonate | _ |
| 622238401 | M05BA07 | Risedronic acid | Bisphosphonate | _ |
| 622239001 | M05BA07 | Risedronic acid | Bisphosphonate | _ |
| 622243601 | M05BA07 | Risedronic acid | Bisphosphonate | _ |
| 622245401 | M05BA07 | Risedronic acid | Bisphosphonate | _ |
| 622245901 | M05BA07 | Risedronic acid | Bisphosphonate | _ |
| 622246001 | M05BA07 | Risedronic acid | Bisphosphonate | _ |
| 622247201 | M05BA07 | Risedronic acid | Bisphosphonate | _ |
| 622248801 | M05BA07 | Risedronic acid | Bisphosphonate | _ |
| 622252601 | M05BA07 | Risedronic acid | Bisphosphonate | _ |
| 622257301 | M05BA07 | Risedronic acid | Bisphosphonate | _ |
| 622258401 | M05BA07 | Risedronic acid | Bisphosphonate | _ |
| 622259901 | M05BA07 | Risedronic acid | Bisphosphonate | _ |
| 622617400 | M05BA07 | Risedronic acid | Bisphosphonate | _ |
| 622688301 | M05BA07 | Risedronic acid | Bisphosphonate | _ |
| 622695101 | M05BA07 | Risedronic acid | Bisphosphonate | _ |
| 622742400 | M05BA07 | Risedronic acid | Bisphosphonate | _ |
| 612320180 | N05AL01 | Sulpiride | Other stomach medicine | _ |
| 612320199 | N05AL01 | Sulpiride | Other stomach medicine | _ |
| 612320200 | N05AL01 | Sulpiride | Other stomach medicine | _ |
| 612320201 | N05AL01 | Sulpiride | Other stomach medicine | _ |
| 612320423 | N05AL01 | Sulpiride | Other stomach medicine | _ |
| 620002520 | N05AL01 | Sulpiride | Other stomach medicine | _ |
| 620003080 | N05AL01 | Sulpiride | Other stomach medicine | _ |
| 620005993 | N05AL01 | Sulpiride | Other stomach medicine | _ |
| 620005994 | N05AL01 | Sulpiride | Other stomach medicine | _ |
| 620006993 | N05AL01 | Sulpiride | Other stomach medicine | _ |
| 620444203 | N05AL01 | Sulpiride | Other stomach medicine | _ |
| 620444206 | N05AL01 | Sulpiride | Other stomach medicine | _ |
| 622733800 | N05AL01 | Sulpiride | Other stomach medicine | _ |
| 622733900 | N05AL01 | Sulpiride | Other stomach medicine | _ |
| 622734000 | N05AL01 | Sulpiride | Other stomach medicine | _ |
| 622734100 | N05AL01 | Sulpiride | Other stomach medicine | _ |

ATC, anatomical therapeutic chemical; WHO, World Health Organization; NSAIDs, non-steroidal anti-inflammatory drugs
