# Supplementary material for: Continuous co-prescription of rebamipide prevents upper gastrointestinal bleeding in NSAID use for orthopaedic conditions: A nested case-control study using the LIFE Study database
Source: PLoS One. 2024 Jun 11;19(6):e0305320. doi: 10.1371/journal.pone.0305320 (PMC11166339; doi:10.1371/journal.pone.0305320)
Supplement: S2 Table — (DOCX) [file pone.0305320.s003.docx]

**S2 Table** Demographics of the cohort in which NSAIDs were first used for osteoarthritis or back pain

| Inclusion and Exclusion criteria and items for interests | Overall  (*n* = 367,714) |
| --- | --- |
| Age at t_0_ | median 75.2  IQR 67–82  range 8–110 |
| Groups of age at t_0_ (no. [%]) |  |
| ≤64 | 71,764 (19.5) |
| 65–74 | 106,365 (28.9) |
| 75–84 | 135,703 (36.9) |
| ≥85 | 53,882 (14.7) |
| Sex at t_0_ (no. [%]) |  |
| Male | 130,165 (35.4) |
| Female | 237,549 (64.6) |
| Diagnosis at t_0_ (no. [%]) |  |
| Osteoarthritis of Hip | 12,396 (3.4) |
| Osteoarthritis of Knee | 121,783 (33.1) |
| Osteoarthritis (Others) | 26,102 (7.1) |
| Back Pain | 207,433 (56.4) |
| Patient with risk drugs of ulcer 90 days before and at t_0_ (no. [%]) |  |
| None | 251,613 (68.4) |
| Antiplatelet agent | 63,198 (17.2) |
| Anticoagulants | 18,965 (5.2) |
| Bisphosphonate | 31,450 (8.6) |
| Steroid | 23,208 (6.3) |
| Upper gastrointestinal endoscopy before and at t_0_ (no. [%]) | 55,072 (15.0) |
| Diagnosis with gastric ulcer or *Helicobacter pylori* before t_0_ (no. [%]) | 87,829 (23.9) |
| NSAIDs at t_0_ |  |
| **Loxoprofen** | 233,575 (63.5) |
| Co-prescription of ulcer prophylaxis (no. [%])  None  Rebamipide  PPI  Misoprostol  H2RAs  Others | 108767 (46.6^a^)  84426 (36.1^a^)  20435 (8.7^a^)  792 (0.3^a^)  9874 (4.2^a^)  21112 (9.0^a^) |
| **Celecoxib** | 91,863 (25.0) |
| Co-prescription of ulcer prophylaxis (no. [%])  None  Rebamipide  PPI  Misoprostol  H2RAs  Others | 34827 (37.9^a^)  37853 (41.2^a^)  10322 (11.2^a^)  368 (0.4^a^)  5352 (5.8^a^)  7514 (8.2^a^) |
| **Diclofenac** | 32,817 (8.9) |
| Co-prescription of ulcer prophylaxis (no. [%])  None  Rebamipide  PPI  Misoprostol  H2RAs  Others | 17525 (53.4^a^)  8592 (26.2^a^)  2293 (7.0^a^)  102 (0.3^a^)  2650 (8.1^a^)  3169 (9.7^a^) |
| **Meloxicam** | 8,961 (2.4) |
| Co-prescription of ulcer prophylaxis (no. [%])  None  Rebamipide  PPI  Misoprostol  H2RAs  Others | 4048 (45.2^a^)  2371 (26.5^a^)  1474 (16.4^a^)  22 (0.2^a^)  667 (7.4^a^)  809 (9.0^a^) |
| **Ibuprofen** | 2,849 (0.8) |
| Co-prescription of ulcer prophylaxis (no. [%])  None  Rebamipide  PPI  Misoprostol  H2RAs  Others | 2030 (71.3^a^)  349 (12.2^a^)  245 (8.6^a^)  1 (0.04^a^)  100 (3.5^a^)  252 (8.8^a^) |

^a^Percentage within the same type of NSAIDs (If multiples prophylaxis was prescribed, the total will be greater than 100%)

NSAIDs, non-steroidal anti-inflammatory drugs; IQR, interquartile range (25^th^ percentile–75^th^ percentile); PPI, proton pump inhibitor; H2RAs, H2 receptor antagonists
