# Supplementary material for: Continuous co-prescription of rebamipide prevents upper gastrointestinal bleeding in NSAID use for orthopaedic conditions: A nested case-control study using the LIFE Study database
Source: PLoS One. 2024 Jun 11;19(6):e0305320. doi: 10.1371/journal.pone.0305320 (PMC11166339; doi:10.1371/journal.pone.0305320)
Supplement: S3 Table — (DOCX) [file pone.0305320.s004.docx]

**S3 Table** Population that received NSAIDs for the first time for osteoarthritis or low back pain and without risk factors for gastric ulcer other than age

| Variables | Cohort  (*n* = 67,565) | Case candidate  from the cohort  (*n* = 345) |
| --- | --- | --- |
| Age at t_0_ (median [IQR]) | 71.2 (63.0–78.5) | 76.5 (68.6–83.9) |
| Groups of age at t_0_ (no. [%]) |  |  |
| ≤64 | 19698 (29.2) | 55 (15.9) |
| 65–74 | 22430 (33.2) | 92 (26.7) |
| 75–84 | 18559 (27.5) | 123 (35.7) |
| ≥85 | 6878 (10.2) | 75 (21.7) |
| Sex at t_0_ (no. [%]) |  |  |
| Male | 24601 (36.4) | 162 (47.0) |
| Female | 42964 (63.6) | 183 (53.0) |
| Rebamipide at t_0_ (no. [%]) | 30635 (45.3) | 149 (43.2) |
| NSAIDs prescribed at t_0_ (no. [%]) |  |  |
| Loxoprofen | 45035 (66.7) | 216 (62.6) |
| Celecoxib | 15499 (22.9) | 102 (29.6) |
| Diclofenac | 5568 (8.2) | 21 (6.1) |
| Meloxicam | 1323 (2.0) | 8 (2.3) |
| Ibuprofen | 490 (0.7) | 1 (0.3) |
| Diagnosis at t_0_ (no. [%]) |  |  |
| Osteoarthritis of Hip | 2274 (3.4) | 13 (3.8) |
| Osteoarthritis of Knee | 20489 (30.3) | 97 (28.1) |
| Osteoarthritis (Others) | 4702 (7.0) | 28 (8.1) |
| Back Pain | 40100 (59.4) | 207 (60.0) |

From the cohort, patients who have undergone gastroscopy procedures and been assigned the ICD-10 of upper gastrointestinal bleeding were considered case candidates

NSAIDs, non-steroidal anti-inflammatory drugs; IQR, interquartile range (25^th^ percentile–75^th^ percentile); ICD-10, International Classification of Disease Tenth Revision
