# Supplementary material for: Continuous co-prescription of rebamipide prevents upper gastrointestinal bleeding in NSAID use for orthopaedic conditions: A nested case-control study using the LIFE Study database
Source: PLoS One. 2024 Jun 11;19(6):e0305320. doi: 10.1371/journal.pone.0305320 (PMC11166339; doi:10.1371/journal.pone.0305320)
Supplement: S10 Table — (DOCX) [file pone.0305320.s011.docx]

**S10 Table** Conditional logistic regression analysis of upper gastrointestinal bleeding (*Sensitivity analysis 3*).

| Prescription status of rebamipide | Case  (*n* = 169) | Control  (*n* = 1,170) | Crude Odds Ratio  (95% CI) | Adjusted Odds Ratio^a^  (95% CI) |
| --- | --- | --- | --- | --- |
| Non-user | 67 (39.6) | 530 (45.3) | 1 (Reference) | 1 (Reference) |
| Continuous-user | 43 (25.4) | 489 (41.8) | 0.70 (0.47–1.04) | 0.67 (0.43–1.04) |
| Irregular-user | 59 (34.9) | 151 (12.9) | 3.09 (2.08–4.58) | 2.93 (1.86–4.60) |

*Sensitivity analysis 3* was performed by adding an exclusion criterion to the main analysis, excluding Charlson Comorbidity Index ≥ 3 point. Consequently, the number of people in the flowchart was replaced by cohort (*n* = 57,805); case candidate (*n* = 277); matched case (*n* = 169); matched control (*n* = 1,170)

^a^Conditional logistic regression analysis was conducted by considering matching factors: (A) sex at t_0_, (B) age (± 5 years) at t_0_, (C) follow-up (± 180 days) from t_0_ to index date, (D) the total number of NSAID tablets prescribed, and (E) the total dose of the five types of NSAIDs (loxoprofen, celecoxib, diclofenac, meloxicam, and ibuprofen)

CI, confidence interval; NSAIDs, non-steroidal anti-inflammatory drugs
