# Supplementary material for: Continuous co-prescription of rebamipide prevents upper gastrointestinal bleeding in NSAID use for orthopaedic conditions: A nested case-control study using the LIFE Study database
Source: PLoS One. 2024 Jun 11;19(6):e0305320. doi: 10.1371/journal.pone.0305320 (PMC11166339; doi:10.1371/journal.pone.0305320)
Supplement: Attachment — Submitted filename: Response to Reviewers.docx [file pone.0305320.s012.docx]

**Journal requirements:**

When submitting your revision, we need you to address these additional requirements.

1. Please ensure that your manuscript meets PLOS ONE's style requirements, including those for file naming. The PLOS ONE style templates can be found at

https://journals.plos.org/plosone/s/file?id=wjVg/PLOSOne_formatting_sample_main_body.pdf and

https://journals.plos.org/plosone/s/file?id=ba62/PLOSOne_formatting_sample_title_authors_affiliations.pdf

**Response: Thanks for the information.**

**We have revised the manuscript according to the PLOS ONE's style requirements.**

2. Please provide additional details regarding participant consent. In the ethics statement in the Methods and online submission information, please ensure that you have specified (1) whether consent was informed and (2) what type you obtained (for instance, written or verbal, and if verbal, how it was documented and witnessed). If your study included minors, state whether you obtained consent from parents or guardians. If the need for consent was waived by the ethics committee, please include this information.

If you are reporting a retrospective study of medical records or archived samples, please ensure that you have discussed whether all data were fully anonymized before you accessed them and/or whether the IRB or ethics committee waived the requirement for informed consent. If patients provided informed written consent to have data from their medical records used in research, please include this information.

**Response: We reconfirm that we had clearly stated in the main text as follows.**

**Text Changes (Materials and methods, Lines 120-122):**

The review board waived the requirement for informed consent due to the study’s retrospective nature and because all records were de-identified and fully anonymized before our access for analysis.

3. Thank you for stating the following financial disclosure:

“This work was supported by the Japan Society for the Promotion of Science KAKENHI (Grant Numbers JP19K21590 and JP20H00563) and by Japan Science and Technology Agency FOREST (Fusion Oriented REsearch for disruptive Science and Technology) program (Grant Number JPMJFR205J).”

Please state what role the funders took in the study. If the funders had no role, please state: "The funders had no role in study design, data collection and analysis, decision to publish, or preparation of the manuscript."

If this statement is not correct you must amend it as needed.

Please include this amended Role of Funder statement in your cover letter; we will change the online submission form on your behalf.

**Response: Please add the following statement to our section of our “Funding”:**

**"The funders had no role in study design, data collection and analysis, decision to publish, or preparation of the manuscript."**

**Text Changes (Funding):**

This work was supported by the Japan Society for the Promotion of Science KAKENHI (Grant Numbers JP19K21590 and JP20H00563) and by Japan Science and Technology Agency FOREST (Fusion Oriented REsearch for disruptive Science and Technology) program (Grant Number JPMJFR205J). The funders had no role in study design, data collection and analysis, decision to publish, or preparation of the manuscript.

4. Thank you for stating the following in the Acknowledgments Section of your manuscript:

“We would like to thank Editage (www.editage.com) for English language editing (funding source: JPMJFR205J).”

We note that you have provided funding information that is currently declared in your Funding Statement. However, funding information should not appear in the Acknowledgments section or other areas of your manuscript. We will only publish funding information present in the Funding Statement section of the online submission form.

Please remove any funding-related text from the manuscript and let us know how you would like to update your Funding Statement. Currently, your Funding Statement reads as follows:

“This work was supported by the Japan Society for the Promotion of Science KAKENHI (Grant Numbers JP19K21590 and JP20H00563) and by Japan Science and Technology Agency FOREST (Fusion Oriented REsearch for disruptive Science and Technology) program (Grant Number JPMJFR205J).”

Please include your amended statements within your cover letter; we will change the online submission form on your behalf.

**Response: We removed the funding-related text from the manuscript.**

**Please revise our “Funding” section as follows.**

**Text Changes (Funding):**

This work was supported by the Japan Society for the Promotion of Science KAKENHI (Grant Numbers JP19K21590 and JP20H00563) and by Japan Science and Technology Agency FOREST (Fusion Oriented REsearch for disruptive Science and Technology) program (Grant Number JPMJFR205J). The funders had no role in study design, data collection and analysis, decision to publish, or preparation of the manuscript.

5. Thank you for stating the following in the Competing Interests section:

“Authors with competing interests

I have read the journal's policy and the authors of this manuscript have the following competing interests:Yasuharu Nakashima received research grants from Chugai Pharmaceutical Co., Ltd., AYUMI Pharmaceutical Corporation, EA Pharma Co., Ltd., KYOCERA Corporation, and Zimmer Biomet G.K. All other authors have no conflicts of interest to disclose.”

Please confirm that this does not alter your adherence to all PLOS ONE policies on sharing data and materials, by including the following statement: "This does not alter our adherence to PLOS ONE policies on sharing data and materials.” (as detailed online in our guide for authors http://journals.plos.org/plosone/s/competing-interests). If there are restrictions on sharing of data and/or materials, please state these. Please note that we cannot proceed with consideration of your article until this information has been declared.

Please include your updated Competing Interests statement in your cover letter; we will change the online submission form on your behalf.

**Response: Please add the following statement to our “Competing Interests” section:**

**"This does not alter our adherence to PLOS ONE policies on sharing data and materials."**

**Text Changes (Competing Interests):**

Yasuharu Nakashima received research grants from Chugai Pharmaceutical Co., Ltd., AYUMI Pharmaceutical Corporation, EA Pharma Co., Ltd., KYOCERA Corporation, and Zimmer Biomet G.K. All other authors have no conflicts of interest to disclose. This does not alter our adherence to PLOS ONE policies on sharing data and materials.

6. We note that you have indicated that there are restrictions to data sharing for this study. PLOS only allows data to be available upon request if there are legal or ethical restrictions on sharing data publicly. For more information on unacceptable data access restrictions, please see http://journals.plos.org/plosone/s/data-availability#loc-unacceptable-data-access-restrictions.

Before we proceed with your manuscript, please address the following prompts:

a) If there are ethical or legal restrictions on sharing a de-identified data set, please explain them in detail (e.g., data contain potentially identifying or sensitive patient information, data are owned by a third-party organization, etc.) and who has imposed them (e.g., a Research Ethics Committee or Institutional Review Board, etc.). Please also provide contact information for a data access committee, ethics committee, or other institutional body to which data requests may be sent.

b) If there are no restrictions, please upload the minimal anonymized data set necessary to replicate your study findings to a stable, public repository and provide us with the relevant URLs, DOIs, or accession numbers. For a list of recommended repositories, please see

https://journals.plos.org/plosone/s/recommended-repositories. You also have the option of uploading the data as Supporting Information files, but we would recommend depositing data directly to a data repository if possible.

We will update your Data Availability statement on your behalf to reflect the information you provide.

**Response: Please revise our Data Availability Statement as follows.**

**Text Change (Data Availability Statement):**

Data Availability Statement: The data used in this study were acquired under agreements between Kyushu University and the participating municipalities, which stipulate that the data can only be used by authorized research institutions and cannot be shared with third parties. However, research institutions that have entered into agreements with the authorized research group in Kyushu University may access the data. Please contact the Joint Research Department of Kyushu University (ijkkyoudou@jimu.kyushu-u.ac.jp) regarding data access.

**Response to Reviewer #1:**

Reviewer #1 (1): Estimated Editor and Authors,

First, I would like to thank you for the opportunity to review this manuscript. This is a nested case-control that evaluated the effectiveness of the prescription of rebamipide for prevention of upper gastrointestinal bleeding in NSAIDs users living with orthopedic conditions. The manuscript is well written and is relevant in the scope of patient safety, considering the high morbidity and mortality related to gastrointestinal bleeding as an adverse drug event. However, methodological reporting is somewhat confusing and needs to be improved for a better understanding of the study and to be reproducible.

Follow my feedback below to help you improve the quality and clarity of your manuscript:

**Response: We are grateful to Reviewer #1 for critical comments and useful suggestions that helped us to improve our paper considerably. We appreciate you pointing out that the description of our methodology was unclear. We did our best effort to revise the manuscript according to your comments.**

Reviewer #1 (2):

- Title: The authors report as if the rebamipide prevents upper gastrointestinal bleeding on any NSAIDS user, but the authors' findings only identified this protection in occasional users. Therefore, it is important to review the title so that it is harmonious with the findings of the study.

**Response:**

**Thank you for your valuable comment. We identified this protection in continuous users. We have revised the title to be appropriate.**

**Text Changes (Title):**

Continuous co-prescription of rebamipide prevents upper gastrointestinal bleeding in NSAID use for orthopaedic conditions: A nested case-control study using the LIFE Study database

Reviewer #1 (3):

- At many times the occasional use of NSAIDS is cited, but this is not defined. It is very important to define how stratified the consumption of NSAIDS and what was considered in each stratum.

**Response: We have modified the wording as we believe Irregular-user would be more appropriate than occasional-user. We also added a definition of grouping to Abstract. In addition, we added a figure to the main text that illustrates the definition of the grouping.**

**Text Changes (Abstract, Lines 36-41):**

Exposure to rebamipide was defined as prescription status from t_0_ to index date: Non-user (rebamipide was not co-prescribed during the follow-up period), Continuous-user (rebamipide was co-prescribed from t_0_ with the same number of tablets as NSAIDs), and Irregular-user (neither Non-user nor Continuous-user). Conditional logistic regression analysis was conducted to estimate each category’s odds ratio compared to non-users.

**Text Changes (Materials and methods, Lines 177-182):**

Patients were grouped based on the prescription status of rebamipide from t_0_ to the index date: 1) Non-user, 2) Continuous-user, and 3) Irregular-user (Fig 1). Patients without rebamipide prescriptions from t_0_ to the index date were grouped as non-users. If patients were continuously prescribed rebamipide from t_0_ to the index date with the same number of tablets as NSAIDs, they were grouped as continuous-users. Patients not falling under either continuous-user or non-user were grouped as irregular-users.

**Text Changes (Fig 1., Lines 184-188):**

**Fig 1. Definition of the prescription status of rebamipide.** Non-user = Rebamipide was not co-prescribed during the follow-up period. Continuous-user = Rebamipide was co-prescribed from t_0_ with the same number of tablets as NSAIDs. Irregular-user = Neither Non-user nor Continuous-user. NSAIDs, non-steroidal anti-inflammatory drugs.

Reviewer #1 (4):

Introduction

- Page 5, Line 60: Is there no more current data on the rebamipide prescription?

**Response:**

**We thank the reviewer for the valuable comment.**

**We have checked the latest data available and updated the numbers.**

**Text Changes (Introduction, Lines 51-53):**

According to the government’s annual all-counts survey, 2.5 billion tablets (0.25 million kg) of rebamipide, marketed by 24 pharmaceutical companies, were prescribed from April 2021 to March 2022.

Reviewer #1 (5):

- The most appropriate terminology is "adverse drug events" and not "effects".

**Response:** **We thank the reviewer for the valuable comment. We agree that the term "adverse drug events" was appropriate and have revised the relevant section.**

**Text Changes (Introduction, Lines 68-69):**

Recently, there have been several reports linking PPIs with adverse drug events,

**Text Changes (Introduction, Lines 72-73):**

On the other hand, rebamipide prevents ulcers without affecting gastric acid secretion[1], is known to have very few adverse drug events,

**Text Changes (Introduction, Lines 284-285):**

They also found that rebamipide was associated with fewer adverse drug events,

**Text Changes (Discussions, Lines 326-327):**

than Caucasians could have an increased risk of adverse drug events associated with PPIs.

**Text Changes (Discussions, Lines 330-331):**

such as Asians having an increased likelihood of experiencing adverse drug events from PPIs,

Reviewer #1 (6):

- Important to define what was considered "low risk of NSAIDs induced ulcers".

**Response: We thank the reviewer for the valuable comment. We listed the risk factors and clarified that patients without these factors were defined as low risk patients.**

**Text Changes (Introduction, Lines 62-68):**

The effectiveness of PPIs in preventing NSAID-induced ulcers has been reported in numerous randomized controlled trials and is also recommended by worldwide guidelines for moderate or high-risk patients who have any of the following risk factors: age over 65 years, previous history of an uncomplicated ulcer, or concurrent use of aspirin, antiplatelet drugs, corticosteroids, or anticoagulant agents. However, their effectiveness in low-risk patients without risk factors remains unclear.

Reviewer #1 (7):

- Page 6, lines 86 and 87 do not fit in the introduction: "We used medical claims data for the analysis we consider it a useful approach for tracking cats across specialties in the real world."

**Response: We thank the reviewer for the valuable comment. We have moved the relevant sentence from Introduction to Methods.**

**Text Changes (Materials and methods, Lines 103-104):**

We used medical claims data for the analysis as we considered it a useful approach for tracking patients across specialties in the real world.

Reviewer #1 (8):

Methodology

- The methodology should follow the reporting of checklist Strobe, because in many moments this section is confused. Strobe should be used to guide the methodology report.

I understand, for example, that the methodology should start by presenting the study and setting design and not the database used to consult the data.

**Response: We have rearranged the method headings and the order of content to follow STROBE whenever possible.**

**Text Changes (Materials and methods, Lines 88-100):**

**Study design**

**Text Changes (Materials and methods, Lines 102-124):**

**Setting**

**Text Changes (Materials and methods, Lines 126-187):**

**Participants**

Reviewer #1 (9):

- Page 6, Line 103: Do two public insurance systems cover the entire country?

**Response: The two public insurance programs cover the majority of the older population, however, do not cover the entire population. Therefore, we added a sentence that specifically indicates the coverage rate.**

**Text Changes (Materials and methods, Lines 113-116):**

The former enrolls 26.3% of all citizens aged 0 to 74, of which 68.5% of all citizens aged 65 to 74 are enrolled, and the latter is in principle enrolled by all citizens aged 75 and older, thus these two insurance policies cover the majority of the elderly in Japan.

Reviewer #1 (10):

- How many researchers conducted data collection? Were pre-delineated forms used for this data collection?

**Response: Five researchers conducted data collection. Yes, pre-delineated forms, specifically the LIFE Common Data Model [H. Fukuda, J Epidemiol 2022] were used for data collection.**

**Text Changes (Materials and methods, Lines 116-119):**

The claims data included recorded diagnoses based on the International Classification of Disease, Tenth Revision (ICD-10) codes, drug prescriptions, and medical treatment during clinical encounters, collected by five researchers using pre-delineated forms of LIFE Common Data Model.

Reviewer #1 (11):

- Why was the Time Window (T.W.) from 90 days before t0 to 1 day before t0 the exclusion criteria?

**Response: We have added a description explaining the reason.**

**Text Changes (Materials and methods, Lines 131-133):**

Considering that insurance payers often do not allow prescriptions to exceed 90 days, therefore, the time window (T.W.) from 90 days before t_0_ to 1 day before t_0_ was used for defining exclusion criteria.

Reviewer #1 (12):

- Were the life habits of patients, such as smoking and consumption of beverages, which depending on the amount consumed may be associated with gastrointestinal problems?

**Response: Because information regarding patient's smoking and consumption of beverages cannot be obtained from our medical receipts, we cannot discuss the association of such life habits of patients with gastrointestinal problems.**

**Text Changes (Discussion, Lines 348-350):**

Second, this study did not adjust for confounding factors that cannot be captured by medical receipt data, such as patients’ smoking and alcohol consumption habits.

Reviewer #1 (13):

- Pages 8 and 9, lines 151 - 153: This sentence is not clear. What refers to censorship in line 153?

**Response: We have added a description to clarify our methodology.**

**Text Changes (Materials and methods, Lines 153-156):**

Patients who received high-risk drugs (anticoagulants, antiplatelet agents, steroids, or bisphosphonates) after t_0_ were censored on that previous day. Therefore, only data from the period until the prescription of high-risk drugs were used for the analysis.

Reviewer #1 (14):

- The definition of cases is not clear. Patients diagnosed with osteoarthritis or back pain who were prescribed from NSAIDs were follow-up until these patients undergoing endoscopy and developed upper gastrointestinal bleeding? This flow of recruitment of participants was not clear to me.

**Response: Thanks for your valuable comments. We have added a description to clarify our methodology. We have added a description of the concept of nested case-control research to help readers understand it.**

**Text Changes (Materials and methods, Lines 159-167):**

In a nested case-control study, cases within the cohort experience the event of interest selected from the follow-up period. In this study, the event of interest was upper gastrointestinal bleeding. Patients were considered as cases if the following two criteria (A) and (B) were met: (A) there was a claim for gastroscopy (treatment code: 160093810) or a hemostatic procedure (treatment code: 150164850) from the day after the first NSAID prescription until 180 days after the last NSAID prescription date within the follow-up period; (B) there was a diagnosis of ‘upper gastrointestinal bleeding’ (ICD-10 codes: K250, K252, K254, K256, K260, K262, K264, K266, K290, and K922) was required in the same calendar month.

Reviewer #1 (15):

- If the patient had in his medical record the reporting of endoscopic findings such as "duodenal ulcer" or "gastric ulcer", would it be considered case? As already reported in the limitations of the study, the non-analysis of endoscopic reports consists of an important bias.

**Response: No, it would not necessarily be considered case, because as we indicated in the method, we did not use the ICD-10 codes of "duodenal ulcer" or "gastric ulcer". We only considered patients possessing a claim for gastroscopy (treatment code: 160093810) or hemostatic procedure (treatment code: 150164850) and who had a diagnosis of “upper gastrointestinal bleeding” (ICD-10 codes: K250, K252, K254, K256, K260, K262, K264, K266, K290, and K922) in the same month.**

**We decided to remove the statement you highlighted in your comment because we felt it could be confusing to readers.**

**Text Changes (Materials and methods, Original Lines 160-167):**

*****(Removed)** We focused on “upper gastrointestinal bleeding” because, in Japanese medical claims, the disease names “gastritis” and “gastric ulcer” are often used interchangeably, [31] probably to maintain consistency in the prescribed drugs. However, we assumed that the fictitious disease name of “upper gastrointestinal bleeding” would be rarely assigned to a patient without any symptoms of gastrointestinal ulcer. Moreover, this diagnosis would never be made without a direct observation of bleeding; therefore, we defined cases as those who also underwent gastroscopy.*******

Reviewer #1 (16):

Another aspect, the intention was to evaluate upper gastrointestinal bleeding as an adverse event of the use of NSAIDS, right? Therefore, this excludes the non-variceal etiology. How was the control of the etiologies of the upper gastrointestinal bleeding? Was it possible to identify if the patient was bleeding by neoplasia, cirrhosis, varicose veins, or other conditions? This aspect needs to be better reported.

**Response: We did not assume upper gastrointestinal bleeding due to etiologies such as neoplasia, cirrhosis, or varicose veins because our analysis was limited to low-risk patients. Moreover, we have also performed a sensitivity analysis that excludes patients having any one disease included in the calculation of the Charlson Comorbidity Index to confirm the robustness of our results.**

**Text Changes (Discussion, Lines 345-348):**

While we found that NSAIDs might unexpectedly cause upper gastrointestinal bleeding, this conclusion is based on studies with low-risk patients. We also have ensured the robustness of our findings by sensitivity analyses, excluding patients with a Charlson Comorbidity Index score of 1 or greater.

Reviewer #1 (17):

- I was also in doubt in the following excerpt "However, we assume that the fictitious disease name of "upper gastrointestinal bleeding" would be rarely assigned to a patent without any symptoms of gastrointestinal ulcer." Was it considered cases only patients with upper with gastrointestinal bleeding or just with ulcer as well? Were evaluated if these patients had signs of recent bleeding?

**Response: All criteria used to determine the case were as written in the Methods. We clarified that the case in this study was an upper gastrointestinal bleeding. We decided to remove the statement you highlighted in your comment because we felt it could be confusing to readers.**

**Text Changes (Materials and methods, Lines 160-161):**

In this study, the event of interest was upper gastrointestinal bleeding.

**Text Changes (Materials and methods, Original Lines 160-167):**

*****(Removed)** We focused on “upper gastrointestinal bleeding” because, in Japanese medical claims, the disease names “gastritis” and “gastric ulcer” are often used interchangeably, [31] probably to maintain consistency in the prescribed drugs. However, we assumed that the fictitious disease name of “upper gastrointestinal bleeding” would be rarely assigned to a patient without any symptoms of gastrointestinal ulcer. Moreover, this diagnosis would never be made without a direct observation of bleeding; therefore, we defined cases as those who also underwent gastroscopy.*******

Reviewer #1 (18):

- Page 10, line 176: define what was considered "1) continuous user, 2) occasional user, and 3) non-user".

**Response: We moved Figures from Supplementary data to the main manuscript and added more detailed descriptions. We have modified the wording as we believe Irregular-user would be more appropriate than occasional-user.**

**Text Changes (Materials and methods, Lines 176-188):**

**Grouping**

**Patients were grouped based on the prescription status of rebamipide from t_0_ to the index date: 1) Non-user, 2) Continuous-user, and 3) Irregular-user (Fig 1). Patients without rebamipide prescriptions from t_0_ to the index date were grouped as non-users. If patients were continuously prescribed rebamipide from t_0_ to the index date with the same number of tablets as NSAIDs, they were grouped as continuous-users. Patients not falling under either continuous-user or non-user were grouped as irregular-users.**

**Fig 1. Grouping based on the prescription status of rebamipide. Non-user = Rebamipide was not co-prescribed during the follow-up period. Continuous-user = Rebamipide was co-prescribed from t_0_ with the same number of tablets as NSAIDs. Irregular-user = Neither Non-user nor Continuous-user. NSAIDs, non-steroidal anti-inflammatory drugs.**

Reviewer #1 (19):

- Page 11, line 206: the following sentence is confusing: "the analytical results are planned to be disseminated to the public through these participating municipalities."

**Response: We thank the reviewer for the valuable comment. We have revised the text.**

**Text Changes (Materials and methods, Lines 212-215):**

However, we shared our study protocol with participating municipalities. In line with the aim of the LIFE Study database, the analytical results are planned to be fed back to the public through these participating municipalities.

Reviewer #1 (20):

- One piece of information that would greatly enrich the results would be to assess the defined daily dose of NSAIDs consumed by the participants. I suggest that the authors assess the feasibility or relevance of including this data.

**Response: We thank the reviewer for the valuable comment. Since the main focus of this study was Rebamipide, the descriptive data on the amount of NSAIDs prescribed only described the total prescriptions used for matching. We have modified Table 1 to indicate the data obtained by excluding 0 mg and to also show the number of individuals. Regarding the amount of daily NSAIDs consumed, future studies are encouraged.**

**Text Changes (Materials and methods, Lines 235-237):**

Total dose of NSAIDs for the cases used for the control matching factor showed a wide distribution in the quartile range for loxoprofen, celecoxib, and diclofenac.

**Text Changes (Table 1, Lines 244-249):**

**Lower part of Table 1. Patient characteristics of cases and controls in this study.**

| Variable | Case (*n* = 215) | Control (*n* = 1,516) |
| --- | --- | --- |
| Total dose of NSAIDs^a,b^ |  |  |
| Loxoprofen (mg) | (n = 155^c^)  median 600  IQR 330–1,260  range 60–18,240 | (n = 1,146^c^)  median 420  IQR 300–840  range 60–18,240 |
| Celecoxib (mg) | (n = 52^c^)  median 1,400  IQR 7,00–2,800  range 100–21,000 | (n = 334^c^)  median 1,400  IQR 700–2,800  range 100–21,000 |
| Diclofenac (mg) | (n = 10^c^)  median 62.5  IQR 31.25–228.12  range 25–525 | (n = 38^c^)  median 50  IQR 25–50  range 25–525 |
| Meloxicam (mg) | (n = 2^c^)  median 70  IQR 70–70  range 70–70 | (n = 5^c^)  median 70  IQR 70–70  range 70–70 |
| Ibuprofen (mg) | (n = 1^c^)  1,500 | (n = 1^c^)  1,500 |
| Total tablets of NSAIDs^a^ (median [IQR, range]) | median 10  IQR 6–23  range 1–304 | median 7  IQR 5–14  range 1–304 |

NSAIDs, non-steroidal anti-inflammatory drugs; IQR, interquartile range (25^th^ percentile–75^th^ percentile)

^c^Number of patients included in the relevant section after excluding patients with 0 mg.

Reviewer #1 (21):

-Was it possible to control whether patients self-medicated with NSAIDs, in addition to the medical prescription they received? How are NSAIDs sold in Japan? In many patients, there is free access to this class.

**Response: In our study, we could not monitor or control the use of NSAIDs that patients may have obtained from sources other than their prescribed medication. In Japan, patients can purchase NSAIDs themselves without restrictions.**

**However, as we stated in our strength, Japan has a universal health insurance system in which most citizens visit hospitals and receive prescriptions for NSAIDs and gastroprotective medicines from their doctors.** **All participants in this study were patients who visited a hospital with insurance. Given that patients are required to pay a maximum of 30% out-of-pocket for prescription drugs, it seems unlikely that they would opt to buy NSAIDs at considerably higher costs on their own.**

**Text Changes (Discussion, Lines 350-356):**

Japan has a universal health insurance system, in which most citizens visit hospitals and receive prescriptions for NSAIDs and gastroprotective medicines from their doctors. Since all individuals in this study visited a hospital and are covered by insurance, paying a maximum of 30% out-of-pocket for prescription medication, we assume that there is a low likelihood they would purchase NSAIDs at significantly higher prices by not using their insurance.

Reviewer #1 (22):

Results

- When reporting averages or medians, I suggest including the standard deviation or quartiles, respectively.

**Response: We thank the reviewer for the valuable comment. Our expression may have been confusing and we have revised it.**

**Text Changes (Table 1, Lines 245-250):**

**Upper part of Table 1. Patient characteristics of cases and controls in this study.**

| Variable | Case (*n* = 215) | Control (*n* = 1,516) |
| --- | --- | --- |
| Index date (range)^a^ | 11 Sep 2013 to  11 Nov 2020 | 11 Sep 2013 to  11 Nov 2020 |
| Days from t_0_ to index^a^ | median 134  IQR 48–377  range 1–1836 | median 159  IQR 76–292  range 1–1656 |
| Age at t_0_^a^ | median 76.2  IQR 69–83  range 36.7–98.5 | median 75.8  IQR 69–81  range 32.6–98.8 |

NSAIDs, non-steroidal anti-inflammatory drugs; IQR, interquartile range (25^th^ percentile–75^th^ percentile)

^a^Variables used for matching

Reviewer #1 (23):

- In table 1, the data presented in the last rows is not clear. Is it the dose of each NSAID and the respective quartiles? Why are most of the drugs set to 0? I think this form of presentation is a bit confusing. I apologize if I'm not understanding the presentation correctly.

**Response: The large number of 0 mg cells was because the matching results only included a large number of patients who were using only loxoprofen. Therefore, we had reported a subgroup analysis by NSAIDs used in t_0_. We have modified Table 1 to indicate the data obtained by excluding 0 mg and to also show the number of individuals.**

**Text Changes (Table 1, Lines 245-250):**

**Lower part of Table 1. Patient characteristics of cases and controls in this study.**

| Variable | Case (*n* = 215) | Control (*n* = 1,516) |
| --- | --- | --- |
| Total dose of NSAIDs^a,b^ |  |  |
| Loxoprofen (mg) | (n = 155^c^)  median 600  IQR 330–1,260  range 60–18,240 | (n = 1,146^c^)  median 420  IQR 300–840  range 60–18,240 |
| Celecoxib (mg) | (n = 52^c^)  median 1,400  IQR 7,00–2,800  range 100–21,000 | (n = 334^c^)  median 1,400  IQR 700–2,800  range 100–21,000 |
| Diclofenac (mg) | (n = 10^c^)  median 62.5  IQR 31.25–228.12  range 25–525 | (n = 38^c^)  median 50  IQR 25–50  range 25–525 |
| Meloxicam (mg) | (n = 2^c^)  median 70  IQR 70–70  range 70–70 | (n = 5^c^)  median 70  IQR 70–70  range 70–70 |
| Ibuprofen (mg) | (n = 1^c^)  1,500 | (n = 1^c^)  1,500 |
| Total tablets of NSAIDs^a^ (median [IQR, range]) | median 10  IQR 6–23  range 1–304 | median 7  IQR 5–14  range 1–304 |

NSAIDs, non-steroidal anti-inflammatory drugs; IQR, interquartile range (25^th^ percentile–75^th^ percentile)

^c^Number of patients included in the relevant section after excluding patients with 0 mg.

Reviewer #1 (24):

Discussion

- As the authors cite the importance of variants in the CYP2C19 gene, which can impact the metabolism of proton pump inhibitors, it is also worth highlighting variants in the CYP2C9 gene, as the *2 and *3 alleles are responsible for the slow metabolism of NSAIDs, which increases the risk of gastrointestinal bleeding.

**Response: We thank the reviewer for the valuable comment. We added a paragraph because we agree that genetic variation in the metabolism of NSAIDs is also important.**

**Text Changes (Discussion, Lines 334-338):**

Forgerini et al. reported variants in the CYP2C9 gene, as the *2 and *3 alleles are responsible for the slow metabolism of NSAIDs, which increases the risk of gastrointestinal bleeding. Zhou et al. reported pronounced regional differences in the distribution of the CYP2C9*2 and *3 alleles, underscoring the importance of genetic variability in the CYP2C9 gene for the efficacy and safety of drugs on a global scale.

Reviewer #1 (25):

- The authors could further highlight the clinical implications and contributions of these findings to clinical practice and patient safety.

**Response: We thank the reviewer for the valuable comment. We have discuss the clinical implications, emphasizing the importance of genetic consideration in the prescription of NSAIDs to enhancing patient safety.**

**Text Changes (Discussion, Lines 338-340):**

When considering the risk of NSAID-induced ulcers in the future, it is essential to consider such genetic backgrounds to enhance patient safety and tailor treatment strategies.

**We thank the reviewer for taking the time to review and provide comments to improve the quality of our paper.**

**Response to Reviewer #2:**

Reviewer #2 (1): This is a worthwhile study as there is a paucity of clinical trial data of rebamipide for this indication.

Methods are sound. Inclusion criteria are quite narrow but this helps to avoid confusing.

**Response: We are grateful to Reviewer #2 for critical comments and useful suggestions that helped us to improve our paper considerably. We did our best effort to revise the manuscript according to your comments.**

Reviewer #2 (2):

The discussion about the adverse effects of PPIs compared to those of rebamipide is not equitable. More types of adverse effects have been reported with PPIs, but they have also been used more widely than rebamipide.

It is necessary to make more mention of the adverse effects of rebapimide. The labels list leukopenia and liver dysfunction as adverse effects; although rare (I checked Malaysian and Philippine labels).

**Response: We agree with your comments. We added a description of the adverse drug events associated with Rebamipide.**

**Text Changes (Materials and methods, Lines 294-296):**

However, prescribers need to be aware of the potential for pulmonary adverse events associated with rebamipide and rare instances of leukopenia, highlighting that it is not without risks.

Reviewer #2 (3):

The results are than continuous use of rebamipide was associated with less GI bleeding than no use, but the occasional use was associated with a higher risk than no use. The conclusion than rebamipide may be more beneficial than PPIs is unsound since the effect of PPIs has not be addressed in this study.

**Response: We agree with your comments. We have removed the last sentence of the conclusion because comparisons with PPI cannot be stated from the data of this study.**

**Text Changes (Conclusions, Original Lines 333-335):**

***(Removed) From a risk-benefit perspective, rebamipide may be more beneficial than PPIs as prophylaxis for NSAID-induced ulcers in patients of Asian descent who have low gastric acid secretion without risk factors other than age.***

Reviewer #2 (5):

Some issues need to be addressed:

In the abstract, it should be specified that the patients are at low risk of NSAIDs induced ulcers.

**Response: We thank the reviewer for the valuable comment. We specified the target population was patients without risk factors of NSAIDs induced ulcers other than age.**

**Text Changes (Abstract, Lines 28):**

without risk factors of NSAID-induced ulcers other than age.

Reviewer #2 (6):

Enteric infections and hypomagnesemia should be mentioned as adverse effects of PPI.

**Response: We thank the reviewer for the valuable comment. We added a description of adverse drug events for PPI regarding enteric infections and hypomagnesemia.**

**Text Changes (Discussion, Lines 313-317):**

Yamashiro et al. reported PPI use is associated with an increased risk of hypomagnesemia, especially in males under 60 years and those with low body-mass index. Several systematic reviews suggest that suppressing gastric acid with PPI leads to bacterial colonization and increased susceptibility to enteric infections.

Reviewer #2 (7):

Line 78: “[Rebamipide] and may be superior to PPIs as a prophylactic agent for patients at low risk of NSAID induced ulcers.” This statement is not supported by a reference.

**Response: We thank the reviewer for the valuable comment. We added a reference and revised the text to be consistent consistent with that description.**

**Text Changes (Introduction, Lines 73-75):**

and could be considered as an alternative to PPIs as a prophylactic agent for patients at low risk of NSAID-induced ulcers[6].

**Reference**

[6] Lee MY, Lee S, Heo KN, Kim WY, Jung SH, Ah YM, et al. Rebamipide as a potential alternative gastroprotective agent to proton pump inhibitor in elderly chronic non-steroidal anti-inflammatory drug users without risk factors. Int J Gen Med. 2022;15:2835-45. doi: 10.2147/IJGM.S353098, PMID 35300126.

Reviewer #2 (8):

Line 80 “The prevention of NSAID-induced ulcers can be achieved from the initial prescription of NSAIDs, such as in an orthopaedic outpatient clinic; however…”

This phrase is strange. From the initial prescription of NSAIDs, prevention is established, it is not yet achieved.

**Response: Thank you for pointing out the problem. We have revised the phrase.**

**Text Changes (Introduction, Lines 76-78):**

The prevention of NSAID-induced ulcers can be established from the initial prescription of NSAIDs, such as in an orthopaedic outpatient clinic; however, several of these prescriptions fail to adhere to the guidelines for preventing ulcers.

Reviewer #2 (9):

The methods should make it clear whether it is a calendar month or a 30-day period.

**Response: Thank you for pointing out points that need clarification. We intended all of them to be calendar months. We have corrected the relevant sections.**

**Text Changes (Materials and methods, Lines 128-130):**

The cohort included patients who were prescribed NSAIDs, including selective cyclooxygenase-2 (COX-2) inhibitors (S1 Table), in the calendar month when the ICD-10 codes for osteoarthritis (M16-19) and back pain (M54) were recorded.

**Text Changes (Materials and methods, Lines 165-167):**

(B) there was a diagnosis of ‘upper gastrointestinal bleeding’ (ICD-10 codes: K250, K252, K254, K256, K260, K262, K264, K266, K290, and K922) was required in the same calendar month.

**Text Changes (Materials and methods, Lines 218-220):**

Of the 367,714 patients who were prescribed NSAIDs (including selective COX-2 inhibitors) and had a diagnosis of osteoarthritis or back pain in the same calendar month, 295,950 (80.5%) patients were aged 65 years or older.

Reviewer #2 (10):

Why is ICD-10 code B98.0 (H. pylori as a cause of diseases classified in other chapters) not used as an exclusion criterion?

**Response:**

**Thank you for pointing out the error. B980 and A048 (H. pylori as a cause of diseases classified in other chapters) should be added. After redoing all analyses, five additional patients were excluded from the cohort. We revised the Figure data and to the descriptive data of the cohort. Those five patients were not included in the case and cohort, resulting in no change in the main analysis.**

**Text Changes (Materials and methods, Lines 137-138):**

diagnosis related to Helicobacter pylori infection (ICD-10 codes: K294, K296, A048, A498, and B980),

**Text Changes (Abstract, Lines 42-43):**

Of 67,561 individuals who met the inclusion criteria, 215 cases and 1,516 controls were selected.

**Text Changes (Results, Lines 224-225):**

After applying the exclusion criteria, 67,561 patients were included in the study cohort (Fig 2).

**Figure Changes (Fig 2)**

**Table Changes (S3 Table)**

Reviewer #2 (11):

Line 138: Anticoagulants are associated with bleeding rather than ulcer risk.

**Response: Thank you for your relevant remarks. We have corrected the description.**

**Text Changes (Materials and methods, Lines 138-141):**

(4) patients who received drugs associated with the risk of gastric ulcer development or associated with bleeding (such as anticoagulants, antiplatelet agents, steroids, or bisphosphonates) prescribed during the T.W.,

Reviewer #2 (12):

Authors should provide unadjusted estimates of the main analysis. (STROBE item 16)

**Response: We modified all tables (Table 2 and S4-10 Table) to show Crude odds ratios that do not account for matching factors and adjusted odds ratios that accounted for matching factors.**

**Text Changes (Table 2, Lines 252-257):**

**Table 2. Conditional logistic regression analysis of upper gastrointestinal bleeding.**

| Prescription status of rebamipide | Case  (*n* = 215) | Control  (*n* = 1,516) | Crude Odds Ratio  (95% CI) | Adjusted Odds Ratio*  (95% CI) |
| --- | --- | --- | --- | --- |
| Non-user | 93 (43.3) | 724 (47.8) | 1 (Reference) | 1 (Reference) |
| Continuous-user | 52 (24.2) | 597 (39.4) | 0.68 (0.47–0.97) | 0.65 (0.44–0.96) |
| Irregular-user | 70 (32.6) | 195 (12.9) | 2.79 (1.97–3.95) | 2.57 (1.73–3.81) |

*Conditional logistic regression analysis was conducted by considering matching factors: (A) sex at t_0_, (B) age (± 5 years) at t_0_, (C) follow-up (± 180 days) from t_0_ to index date, (D) the total number of NSAID tablets prescribed, and (E) the total dose of the five types of NSAIDs (loxoprofen, celecoxib, diclofenac, meloxicam, and ibuprofen).

CI, confidence interval; NSAIDs, non-steroidal anti-inflammatory drugs

Reviewer #2 (12):

Line 162: “However, we assumed that the fictitious disease name…”

The phrase is strange. The name is not fictitious.

**Response: All criteria used to determine the case were as written in the Methods. We clarified that the case in this study was an upper gastrointestinal bleeding. We decided to remove the statement you highlighted in your comment because we felt it could be confusing to readers.**

**Text Changes (Materials and methods, Original Lines 160-167):**

***(Removed) We focused on “upper gastrointestinal bleeding” because, in Japanese medical claims, the disease names “gastritis” and “gastric ulcer” are often used interchangeably, [31] probably to maintain consistency in the prescribed drugs. However, we assumed that the fictitious disease name of “upper gastrointestinal bleeding” would be rarely assigned to a patient without any symptoms of gastrointestinal ulcer. Moreover, this diagnosis would never be made without a direct observation of bleeding; therefore, we defined cases as those who also underwent gastroscopy.***

Reviewer #2 (13):

Line 170: the total number of NSAID tablets “prescribed” instead of “administered”.

**Response: Thank you for your appropriate remarks. The term "prescribed" is more appropriate than "administered" in this study, thus we have reviewed the entire paper and corrected the relevant sections.**

**Text Changes (Materials and methods, Lines 95):**

such as the type and dosage of NSAIDs prescribed,

**Text Changes (Materials and methods, Lines 99-100):**

, which could incorporate the information on the dosage and type of NSAIDs prescribed,

**Text Changes (Materials and methods, Lines 173):**

(D) the total number of NSAID tablets prescribed,

**Text Changes (Materials and methods, Lines 363-364):**

, which could strictly adjust the type and dosage of NSAIDs prescribed.

Reviewer #2 (14):

Line 320: “the study sample was limited to low-risk patients”. This is not a limitation since the research question was about low risk patients.

**Response: Thank you for the suggestion. We removed the relevant sentence.**

**Text Changes (Discussion, Original Lines 320):**

*****(Removed) Second, the study sample was limited to low-risk patients.***

Reviewer #2 (15):

S2 Figure The duration of upper gastrointestinal bleeding after the first prescription of NSAIDs

Figure title is wrong. Data are days to bleeding.

**Response: Thank you for pointing out the problem. We have revised the title.**

**Text Changes (S1 Fig. Title, Lines 581):**

**S1 Fig. Days from t_0_ to upper gastrointestinal bleeding.**

**We thank the reviewer for taking the time to review and provide comments to improve the quality of our paper.**
