# Supplementary material for: Continuous co-prescription of rebamipide prevents upper gastrointestinal bleeding in NSAID use for orthopaedic conditions: A nested case-control study using the LIFE Study database
Source: PLoS One. 2024 Jun 11;19(6):e0305320. doi: 10.1371/journal.pone.0305320 (PMC11166339; doi:10.1371/journal.pone.0305320)
Supplement: Attachment — Submitted filename: Response to Reviewers.docx [file pone.0305320.s013.docx]

**Response to Journal requirements:**

Please review your reference list to ensure that it is complete and correct. If you have cited papers that have been retracted, please include the rationale for doing so in the manuscript text, or remove these references and replace them with relevant current references. Any changes to the reference list should be mentioned in the rebuttal letter that accompanies your revised manuscript. If you need to cite a retracted article, indicate the article’s retracted status in the References list and also include a citation and full reference for the retraction notice.

**Response:**

**Thanks for the information. We re-searched all the articles on Pubmed and confirmed that they had not been Retracted.**

**We have added one additional citation (***Sato T, Yamate S, Utsunomiya T, Inaba U, Ike H, Kinoshita K, et al. Life Course Epidemiology of Hip Osteoarthritis in Japan: A Multicenter, Cross-Sectional Study. J Bone Joint Surg Am. 2024; Forthcoming. doi: 10.2106/JBJS.23.01044, PMID 38626018.***) and have indicated accordingly at the end of this letter.**

**Response to Reviewer #1:**

Reviewer #1: Dear Editor and Authors,

Firstly, I thank you for the opportunity to review again this manuscript.

The authors carried out a carefully review and attended all my suggestions. Hence, I am delighted with the revised version and have no other suggestions.

Minor comment: genes should be written in italics.

Finally, I congratulate the authors for their careful review.

**Response:**

**We are grateful to Reviewer #1 for taking the time to review and provide comments to improve the quality of our paper. As commented to us, we have modified the genes to italics.**

**Text Changes (Discussion, Lines 338-345):**

Forgerini et al. reported variants in the ***CYP2C9*** gene, as the ****2*** and ****3*** alleles are responsible for the slow metabolism of NSAIDs, which increases the risk of gastrointestinal bleeding. Zhou et al. reported pronounced regional differences in the distribution of the ***CYP2C9*2*** and ****3*** alleles, underscoring the importance of genetic variability in the ***CYP2C9*** gene for the efficacy and safety of drugs on a global scale. When considering the risk of NSAID-induced ulcers in the future, it is essential to consider such genetic backgrounds to enhance patient safety and tailor treatment strategies.

**Response to Reviewer #2:**

Reviewer #2 (1):

Continuous-user is defined as one whom rebamipide was co-prescribed from t0 with the same number of tablets as NSAIDs. However, as dosage of rebamipide is one tablet 3 times/day and dosage of celecoxib is one tablet/day, the same number of tablets covers fewer days with rebamipide than with celecoxib. This not fit with figure 1.

In which group is someone who is prescribed more rebamipide tablets than NSAIDs (as they should be in the case of celecoxib or meloxicam for being a continuous user)? It is necessary to clarify this issue.

**Response:**

**Thank you for your comment. You are correct in noting that rebamipide is recommended to be taken three times per day, while celecoxib is usually taken twice per day in Japan. We believe that the clinical significance of our results lies in showing that a prescribing pattern where rebamipide and NSAIDs taken with the same number of tablets can be effective. This approach may enhance patient compliance in real-world settings because complex prescribing patterns may reduce patient compliance in real-world clinical practice. We have revised the manuscript to clarify this issue. Thank you for your constructive feedback.**

**Text Changes (Materials and methods, Lines 182-185):**

**Therefore, if a patient was prescribed NSAIDs less than three times a day but rebamipide three times a day [4], they were classified as an irregular user because the number of tablets did not match.**

**Text Changes (Discussion, Lines 275-277):**

**Using rebamipide for preventing NSAID-induced ulcers, a simple co-prescription pattern for rebamipide may improve adherence in clinical practice.**

Reviewer #2 (2):

I am sorry, but I disagree with reviewer #1. “Adverse drug event” is not a standard term; I consider “adverse effect” or “adverse drug reaction” more accurate terms because a causal relationship between the drug and an occurrence is suspected.

Please see: European Medicines Agency and Heads of Medicines Agencies, 2017. Guideline on good pharmacovigilance practices (GVP) Annex I - Definitions (Rev 4). https://www.ema.europa.eu/en/documents/scientific-guideline/guideline-good-pharmacovigilance-practices-annex-i-definitions-rev-4_en.pdf

**Response:**

**Thank you for the information. We have confirmed the PDFs and corrected the terminology to “adverse drug reaction”. Thank you for your careful review to improve the quality of our paper.**

**Text Changes (Introduction, Lines 68-71):**

Recently, there have been several reports linking PPIs with **adverse drug reactions**, such as dementia, bone fractures, prosthetic joint infection, and severe clinical outcomes of coronavirus disease (COVID-19).

**Text Changes (Introduction, Lines 72-75):**

On the other hand, rebamipide prevents ulcers without affecting gastric acid secretion, is known to have very few **adverse drug reactions**, and could be considered as an alternative to PPIs as a prophylactic agent for patients at low risk of NSAID-induced ulcers.

**Text Changes (Discussion, Lines 287-299):**

Rebamipide was comparable to misoprostol in reducing the incidence of gastric and duodenal ulcers according to a multicenter, double-blind, randomized parallel-group study by Kim et al. They also found that rebamipide was associated with fewer **adverse drug reactions**, such as nausea and diarrhea. However, our study found that the actual number of prescriptions is approximately 1/100th of that for rebamipide; this could be because rebamipide has fewer **adverse drug reactions** than misoprostol. Recently, Lee et al. reported no significant difference in the risk of serious gastrointestinal **adverse drug reactions** between PPI and rebamipide for preventing NSAID-induced ulcers (adjusted hazard ratio 0.69, 95% CI 0.27–1.76) using the national claims database. Even though the frequency is lower compared to other acid-related disorder treatments, prescribers should be aware of the risk of pulmonary **adverse drug reactions** associated with rebamipide, and leukopenia might occur in rare cases, meaning that rebamipide is not entirely risk-free.

**Text Changes (Discussion, Lines 330-332):**

Asians with low CYP2C19 levels and lower stomach acid secretion than Caucasians could have an increased risk of **adverse drug** **reactions** associated with PPIs.

**Text Changes (Discussion, Lines 334-337):**

In addition to the effects of unadjusted confounding factors, racial differences, such as Asians having an increased likelihood of experiencing **adverse drug** **reactions** from PPIs, could be related to the discrepancy in results.

Reviewer #2 (3):

Lines 292-296

There is a duplicity:

prescribers should be aware of the risk of pulmonary adverse drug events associated with rebamipide [44], and leukopenia might occur in rare cases [5], meaning that rebamipide is not entirely risk-free. However, prescribers need to be aware of the potential for pulmonary adverse events associated with rebamipide [44] and rare instances of leukopenia [5], highlighting that it is not without risks.

**Response:**

**We are grateful for the careful review of Reviewer #2. We have removed the duplicate sections of the latter.**

**Text Changes (Discussion, Lines 296-299):**

Even though the frequency is lower compared to other acid-related disorder treatments, prescribers should be aware of the risk of pulmonary adverse drug reactions associated with rebamipide [44], and leukopenia might occur in rare cases [5], meaning that rebamipide is not entirely risk-free. ~~However, prescribers need to be aware of the potential for pulmonary adverse events associated with rebamipide [44] and rare instances of leukopenia [5], highlighting that it is not without risks.~~

Reviewer #2 (4):

Line 323

"Using PPIs to prevent NSAID-induced ulcers in patients with no risks should be discussed"

"with no risk factors" or "at low risk" are better expressions.

**Response:**

**Thank you for your comment. We have revised our expressions.**

**Text Changes (Discussion, Lines 326-328):**

Using PPIs to prevent NSAID-induced ulcers in patients **at low risk** should be discussed in light of these possibilities.

Reviewer #2 (5):

Lines 345 346

"While we found that NSAIDs might unexpectedly cause upper gastrointestinal bleeding, this conclusion is based on studies with low-risk patients."

I do not understand the sentence. NSAIDs, expectedly, cause upper gastrointestinal bleeding even in low-risk patients. This is a finding from the present study. What studies are the authors referring to?

**Response: Thank you for your comment. In response to the previous comment by Reviewer #1, our intention was not well expressed.**

**(Previous comment by Reviewer #1:** Another aspect, the intention was to evaluate upper gastrointestinal bleeding as an adverse event of the use of NSAIDS, right? Therefore, this excludes the non-variceal etiology. How was the control of the etiologies of the upper gastrointestinal bleeding? Was it possible to identify if the patient was bleeding by neoplasia, cirrhosis, varicose veins, or other conditions? This aspect needs to be better reported.**)**

**Text Changes (Materials and methods, Lines 350-354):**

Second, we did not assume that upper gastrointestinal bleeding was due to etiologies other than NSAID-induced ulcers. Therefore, we ensured the robustness of our findings by conducting sensitivity analyses that excluded patients with a Charlson Comorbidity Index score of 1 or higher to support the assumption that the upper gastrointestinal bleeding was not due to other causes, such as neoplasia, cirrhosis, or varicose veins.

**Note: We have added one additional citation.**

**Text Changes (Materials and methods, Lines 322-325):**

**According to a multicenter, cross-sectional study conducted in 2022 in Japan, the mean age of new patients with hip osteoarthritis was 63.5 years [53],** and those patients with osteoarthritis may undergo joint replacement surgery after taking NSAIDs for an extended duration.

**Additional Reference:**

**[53] Sato T, Yamate S, Utsunomiya T, Inaba U, Ike H, Kinoshita K, et al. Life Course Epidemiology of Hip Osteoarthritis in Japan: A Multicenter, Cross-Sectional Study. J Bone Joint Surg Am. 2024; Forthcoming. doi: 10.2106/JBJS.23.01044, PMID 38626018.**

**We thank Reviewer #1 and Reviewer #2 for the thorough review of our work, and provide comments to improve the quality of our paper.**
