# Supplementary material for: Continuous co-prescription of rebamipide prevents upper gastrointestinal bleeding in NSAID use for orthopaedic conditions: A nested case-control study using the LIFE Study database
Source: PLoS One. 2024 Jun 11;19(6):e0305320. doi: 10.1371/journal.pone.0305320 (PMC11166339; doi:10.1371/journal.pone.0305320)
Supplement: Attachment — Submitted filename: Response to Reviewers.docx [file pone.0305320.s014.docx]

**Response to Journal requirements:**

Journal Requirements: Please review your reference list to ensure that it is complete and correct. If you have cited papers that have been retracted, please include the rationale for doing so in the manuscript text, or remove these references and replace them with relevant current references. Any changes to the reference list should be mentioned in the rebuttal letter that accompanies your revised manuscript. If you need to cite a retracted article, indicate the article’s retracted status in the References list and also include a citation and full reference for the retraction notice.

**Response:**

**Thank you for the information. We re-searched all the articles on Pubmed and confirmed that they are complete and correct, and none have been retracted.**

**Response to Reviewer #2:**

Reviewer #2: Line 240.

I think what is meant is “The median time to upper gastrointestinal bleeding after the first prescription of NSAIDs was 134 days (S2 Figure).”

**Response:**

**Thank you so much for pointing out the error. Your revised description is correct. We also corrected the numbering of the figure; S1 is correct, not S2.**

**Text Changes (Results, Lines240-242):**

The median **time to** upper gastrointestinal bleeding after the first prescription of NSAIDs was 134 days (**S1** Figure).

**We thank Reviewer #1 and Reviewer #2 for the thorough review of our work, and provide comments to improve the quality of our paper.**
